# Supplementary material for: Bacterial RNA promotes proteostasis through inter-tissue communication in C. elegans
Source: Nat Commun. 2025 Oct 1;16:8650. doi: 10.1038/s41467-025-63987-x (PMC12488917; doi:10.1038/s41467-025-63987-x)
Supplement: Supplementary file 1 — Supplementary Information [file 41467_2025_63987_MOESM1_ESM.pdf]

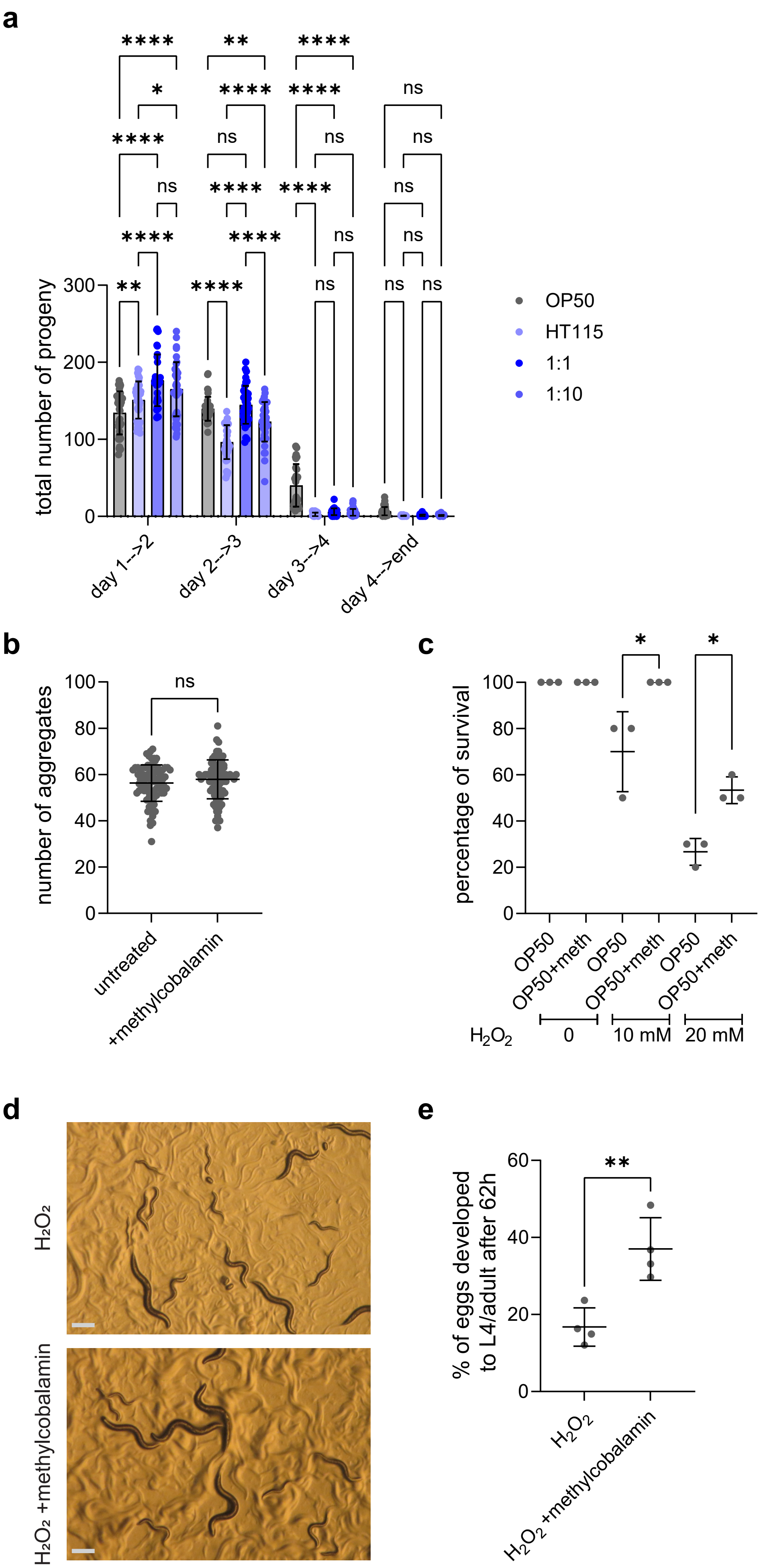

a
total number of progeny
day 1-->2
day 2-->3
day 3-->4
day 4-->end
OP50
HT115
1:1
1:10
b
number of aggregates
untreated
+methylcobalamin
c
percentage of survival
OP50
OP50+meth
H2O2
0
10 mM
20 mM
d
H₂O₂
H₂O₂ +methylcobalamin
e
% of eggs developed to L4/adult after 62h
H2O2
H2O2+methylcobalamin

Supplementary Figure 1. Diet influences reproduction rate of *C. elegans* whereas methylcobalamin supplementation does not influence protein aggregation (a) Brood size of wt worms on different bacterial lawns. The number of progeny per worm per day is shown. Number of worms, *n* = 40 for all conditions. Values represent mean ± SD from four independent experiments. 2way ANOVA with Tukey's multiple comparison test was used. For day 1-->2 OP50 vs. HT115 P = 0.0014, OP50 vs. 1:1 P < 0.0001, OP50 vs. 1:10 P < 0.0001, HT115 vs. 1:1 P < 0.0001, HT115 vs. 1:10 P = 0.0111, 1:1 vs. 1:10 P = 0.512, for day 2-->3 OP50 vs. HT115 P < 0.0001, OP50 vs. 1:1 P = 0.6823, OP50 vs. 1:10 P = 0.0012, HT115 vs. 1:1 P < 0.0001, HT115 vs. 1:10 P < 0.0001, 1:1 vs. 1:10 P < 0.0001, for day 3-->4 OP50 vs. HT115 P < 0.0001, OP50 vs. 1:1 P < 0.0001, OP50 vs. 1:10 P < 0.0001, HT115 vs. 1:1 P = 0.8684, HT115 vs. 1:10 P = 0.9294, 1:1 vs. 1:10 P = 0.9985, for day 4-->end OP50 vs. HT115 P = 0.4930, OP50 vs. 1:1 P = 0.5879, OP50 vs. 1:10 P = 0.5667, HT115 vs. 1:1 P = 0.9988, HT115 vs. 1:10 P = 0.9994, 1:1 vs. 1:10 P > 0.9999. (b) Quantification of polyQ40::YFP fluorescent foci of 2-day old worm cultured on OP50 bacterial lawns supplemented or not with methylcobalamin. The number of aggregates per worm is shown. Number of worms, *n* = 80 (untreated) , *n* = 76 (+methylcobalamin). P = 0.2631. (c) Survival of wt worms on OP50 supplemented or not with methylcobalamin (meth) in the presence of H_2O_2 (0, 10mM or 20mM) was measured. Number of worms, *n* = 30 (OP50), *n* = 30 (OP50 +meth), *n* = 30 (OP50 +H_2O_2 10mM), *n* = 30 (OP50 +meth +H_2O_2 10mM), *n* = 30 (OP50 +H_2O_2 20mM), *n* = 30 (OP50 +meth +H_2O_2 20mM). OP50 vs. OP50+meth 10 mM P = 0.0148, OP50 vs. OP50+meth 20 mM P = 0.0153 (d) Representative images of wt worms on OP50 supplemented or not with methylcobalamin in the presence of 10mM H_2O_2. (e) Quantification of the developmental rate of wt worms on OP50 supplemented or not with methylcobalamin in the presence of 10mM H_2O_2, was measured as the percentage of worms that developed into L4/adult stages after 62 hours. Number of worms, *n* = 1058 (H_2O_2), *n* = 1886 (H_2O_2 +methylcobalamin). P = 0.0036. Values represent mean ± SD from three independent experiments. Mann Whitney t-test (b) or paired t-test (c, e) were used. ns P>0.05, * P<0.05, ** P<0.01. Scale bars are 200µm. Source data are provided as a Source Data file.

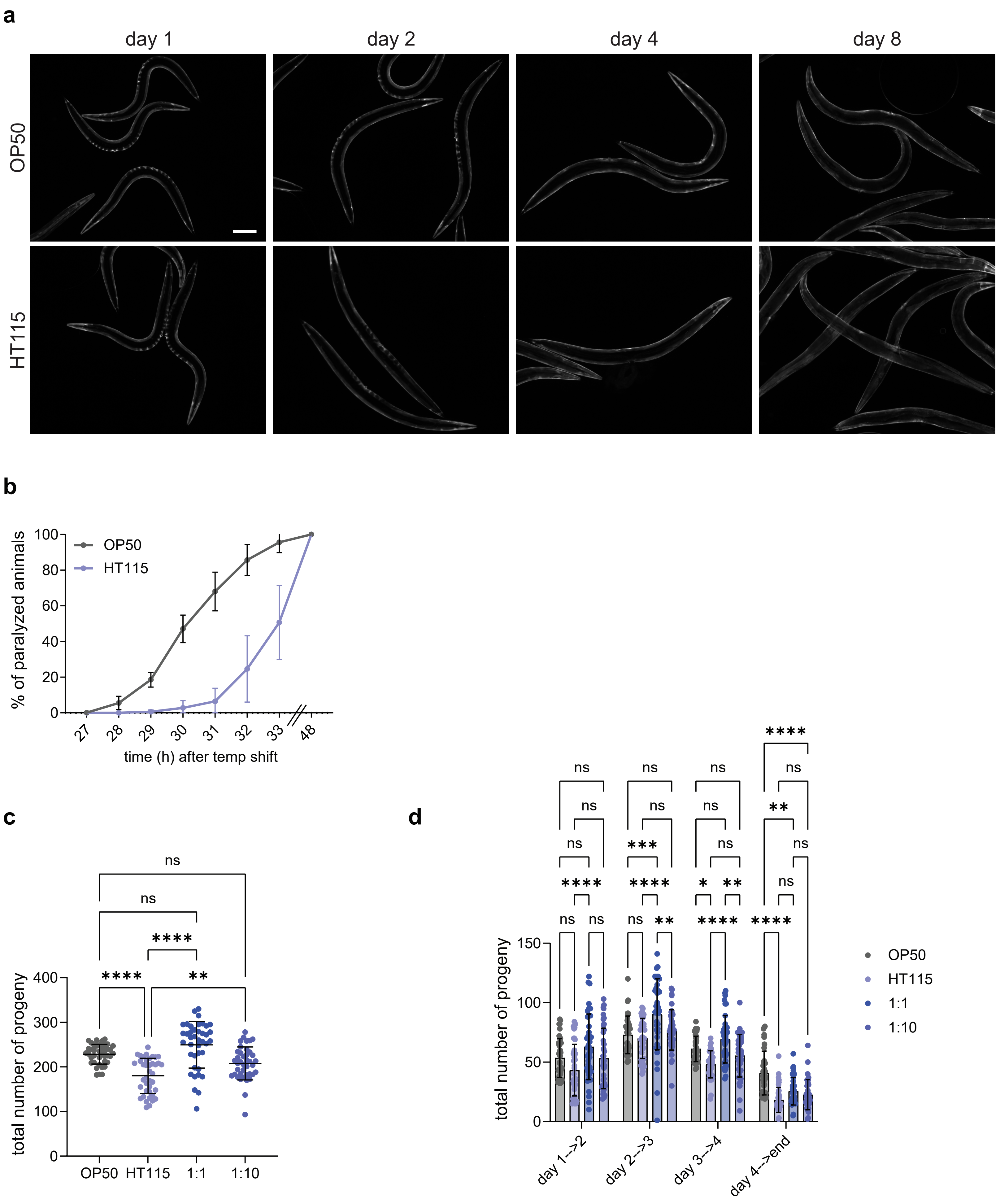

a
day 1
day 2
day 4
day 8
OP50
HT115
b
% of paralyzed animals
OP50
HT115
0
20
40
60
80
100
27
28
29
30
31
32
33
48
time (h) after temp shift
c
total number of progeny
0
100
200
300
400
OP50
HT115
1:1
1:10
ns
ns
****
****
**
d
total number of progeny
0
50
100
150
day 1-->2
day 2-->3
day 3-->4
day 4-->end
OP50
HT115
1:1
1:10
ns
****
***
*
**

Supplementary Figure 2. Dietary effects on amyloid-β-induced paralyses and reproduction of proteotoxically challenged *C. elegans*. (a) Representative images of polyQ24::YFP-expressing worms on OP50 and HT115 during ageing. (b) Paralyses assay of amyloid-β-expressing worms. The percentage of paralysed worms after temperature upshift is shown for worms on OP50 and HT115. Number of worms, n = 968 (OP50), n = 1022 (HT115). (c) Brood size of polyQ40::YFP-expressing worms on different bacterial lawns. The total number of progeny per worm is shown. OP50 vs. HT115 P = 0.0658, OP50 vs. 1:1 P = 0.1118, OP50 vs. 1:10 P = 0.9997, HT115 vs. 1:1 P < 0.0001, HT115 vs. 1:10 P = 0.0833, 1:1 vs. 1:10 P = 0.0895. (d) Brood size of poly-Q40::YFP-expressing worms on different bacterial lawns. The number of progeny per worm per day is shown. Number of worms, n = 40 for all conditions. For day 1-->2 OP50 vs. HT115 P = 0.0014, OP50 vs. 1:1 P < 0.0001, OP50 vs. 1:10 P < 0.0001, HT115 vs. 1:1 P < 0.0001, HT115 vs. 1:10 P = 0.0111, 1:1 vs. 1:10 P = 0.512, for day 2-->3 OP50 vs. HT115 P = 0.8894, OP50 vs. 1:1 P = 0.0002, OP50 vs. 1:10 P = 0.7419, HT115 vs. 1:1 P < 0.0001, HT115 vs. 1:10 P = 0.3078, 1:1 vs. 1:10 P = 0.0099, for day 3-->4 OP50 vs. HT115 P = 0.0107, OP50 vs. 1:1 P = 0.2116, OP50 vs. 1:10 P = 0.5133, HT115 vs. 1:1 P < 0.0001, HT115 vs. 1:10 P = 0.3109, 1:1 vs. 1:10 P = 0.0052, for day 4-->end OP50 vs. HT115 P < 0.0001, OP50 vs. 1:1 P = 0.0015, OP50 vs. 1:10 P < 0.0001, HT115 vs. 1:1 P = 0.3109, HT115 vs. 1:10 P = 0.7349, 1:1 vs. 1:10 P = 0.8966. Values represent mean ± SD from at least three independent experiments. One-way ANOVA with Sidak's multiple comparison test (c) and 2way ANOVA with Tukey's multiple comparison test (d) were used. ns P>0.05, * P<0.05, ** P<0.01, *** P<0.001, **** P<0.0001. Source data are provided as a Source Data file.

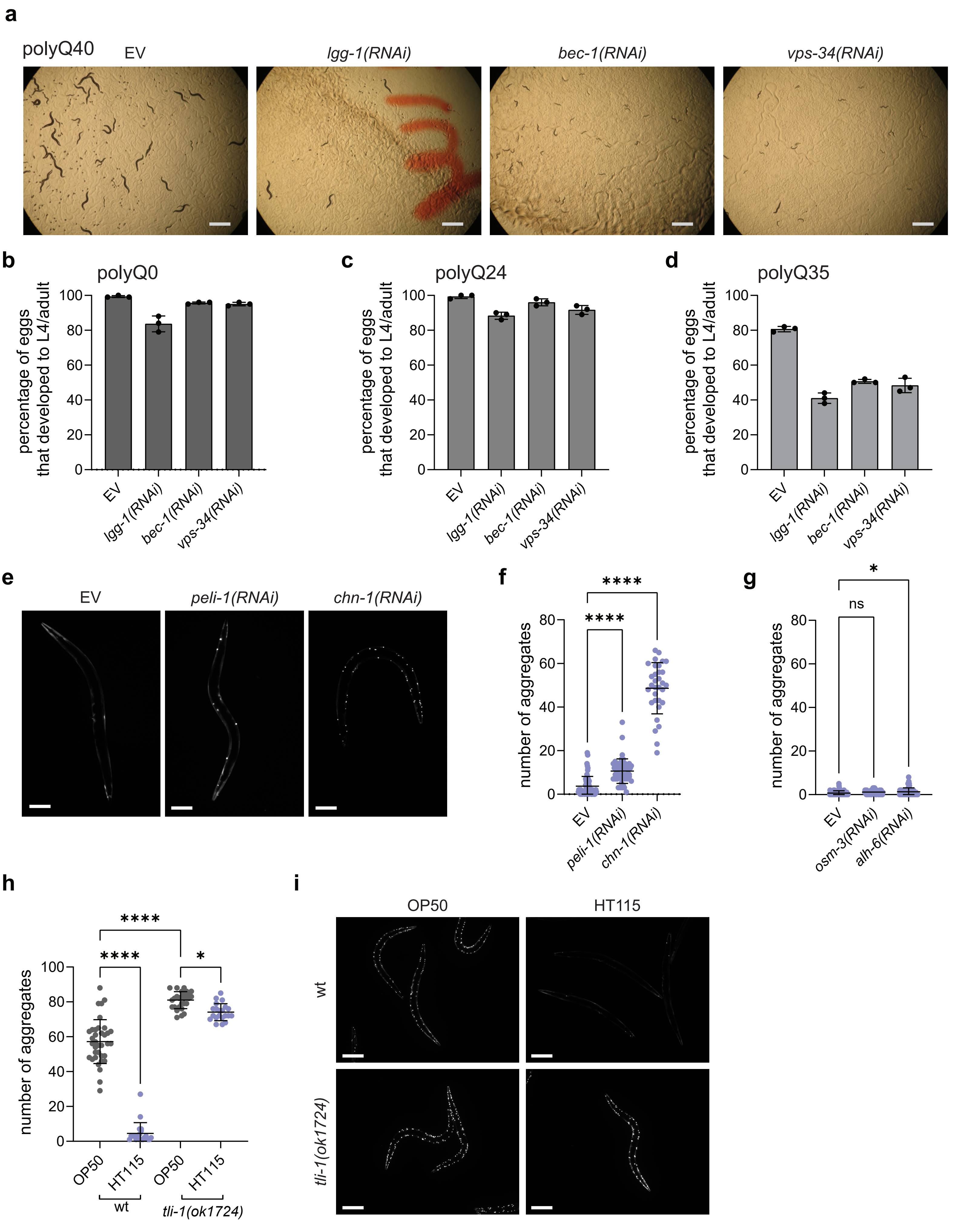

a
polyQ40
EV
lgg-1(RNAi)
bec-1(RNAi)
vps-34(RNAi)
b
polyQ0
c
polyQ24
d
polyQ35
percentage of eggs that developed to L4/adult
0
20
40
60
80
100
EV
lgg-1(RNAi)
bec-1(RNAi)
vps-34(RNAi)
e
EV
peli-1(RNAi)
chn-1(RNAi)
f
****
****
number of aggregates
EV
peli-1(RNAi)
chn-1(RNAi)
g
*
ns
number of aggregates
EV
osm-3(RNAi)
alh-6(RNAi)
h
****
****
*
number of aggregates
OP50
HT115
OP50
HT115
wt
tli-1(ok1724)
i
OP50
HT115
wt
tli-1(ok1724)

Supplementary Figure 3. Autophagy disruption increases protein aggregation and leads to developmental defects of stressed worms. (a) Representative images of polyQ40::YFP-expressing worms on HT115, treated with empty vector (EV), *lgg-1(RNAi), bec-1(RNAi)* and *vps-34(RNAi)*. Scale bar is 1mm. (b-d) Developmental rate of wt (polyQ0) (b), polyQ24::YFP-expressing (c) and polyQ35-expressing (d) worms on HT115 bacterial diet, treated with EV, *lgg-1(RNAi), bec-1(RNAi)* and *vps-34(RNAi)* was measured as the percentage of eggs that developed into L4/adult stages 48h after egg laying. Number of worms *n* = 165 (EV), *n* = 162 (*lgg-1(RNAi)*), *n* = 156 (*bec-1(RNAi)*), *n* = 132 (*vps-34(RNAi)*) for (b), *n* = 202 (EV), *n* = 168 (*lgg-1(RNAi)*), *n* = 184 (*bec-1(RNAi)*), *n* = 135 (*vps-34(RNAi)*) for (c) and *n* = 54 (EV), *n* = 26 (*lgg-1(RNAi)*), *n* = 44 (*bec-1(RNAi)*), *n* = 22 (*vps-34(RNAi)*) for (d). (e) Representative images of polyQ40::YFP-expressing worms on HT115 bacterial diet, treated with EV, *peli-1(RNAi)* and *chn-1(RNAi)*. Scale bar is 100μm. (f) Quantification of polyQ40::YFP fluorescent foci of 2-day old worms on HT115, treated with EV, *peli-1(RNAi)* and *chn-1(RNAi)*. P < 0.0001. The number of aggregates per worm is shown. Number of worms, *n* = 64 (EV), *n* = 50 (*peli-1(RNAi)*), *n* = 54 (*chn-1(RNAi)*). (g) Quantification of polyQ40::YFP fluorescent foci of 2-day old worms on HT115, treated with EV, *osm-3(RNAi)* and *alh-6(RNAi)*. The number of aggregates per worm is shown. Number of worms, *n* = 61 (EV), *n* = 48 (*osm-3(RNAi)*), *n* = 57 (*alh-6(RNAi)*). EV vs. *osm-3(RNAi)* P = 0.2068, EV vs. *alh-6(RNAi)* P = 0.0385. (h) Quantification of polyQ40::YFP fluorescent foci of 2-day old wt and *tli-1(ok1724)* worms on OP50 and HT115. The number of aggregates per worm is shown. Number of worms, *n* = 36 (OP50), *n* = 20 (HT115) for wt and *n* = 28 (OP50), *n* = 21 (HT115) for *tli-1(ok1724)*. OP50 vs. HT115 (wt) P < 0.0001, OP50 (wt) vs. OP50 (*tli-1(ok1724)* P < 0.0001, OP50 vs HT115 (*tli-1(ok1724) P = 0.0183.* (i) Representative images of polyQ40::YFP-expressing wt and *tli-1(ok1724)* mutant worms on OP50 and HT115. Scale bar is 200μm. Values represent mean ± SD. One-way ANOVA with Sidak's multiple comparison test was used. ns P>0.05, * P<0.05, ** P<0.01, *** P<0.001, **** P<0.0001. Source data are provided as a Source Data file.

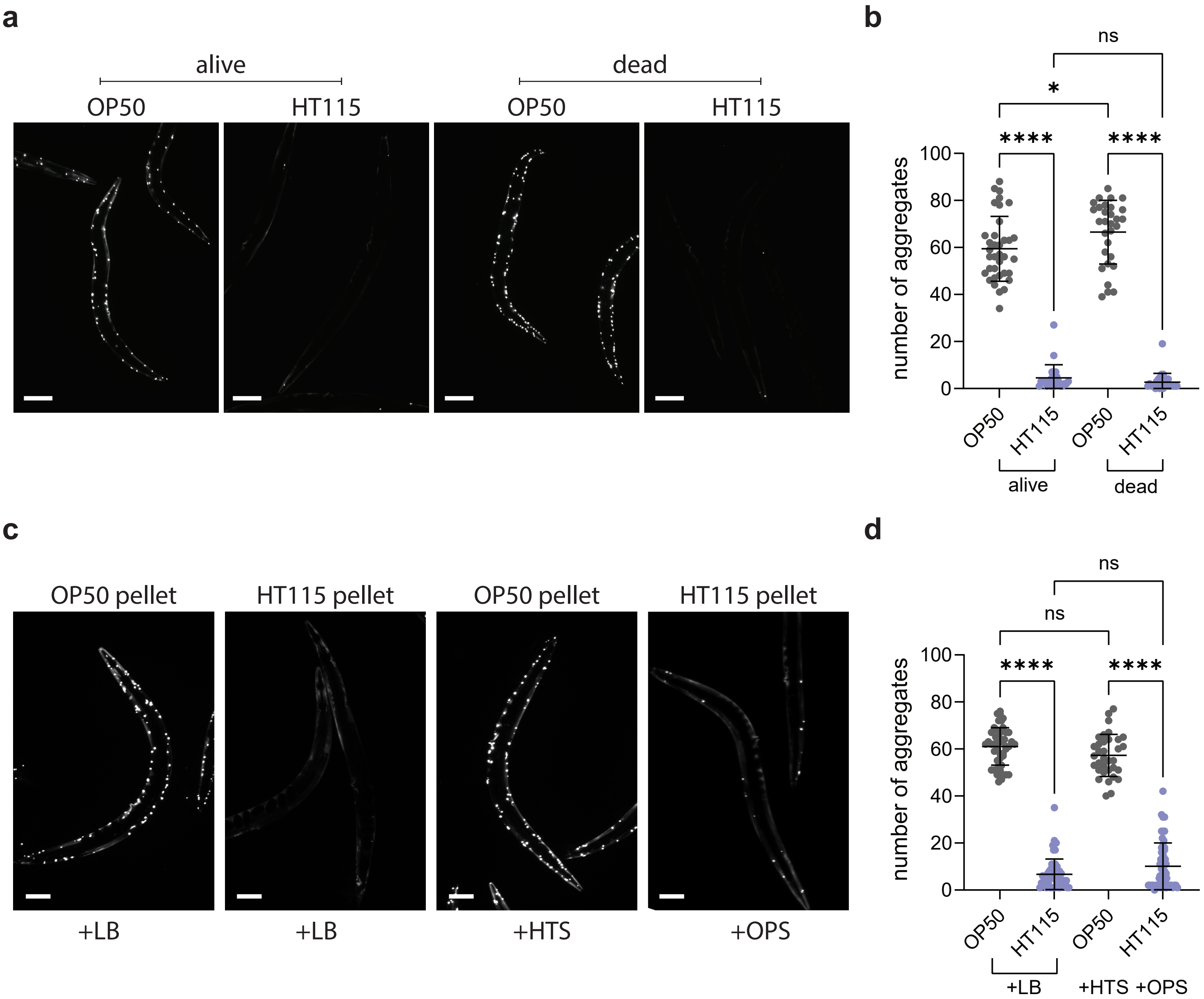
a
alive
dead
OP50
HT115
OP50
HT115
b
ns
*
****
****
number of aggregates
100
80
60
40
20
0
OP50
HT115
OP50
HT115
alive
dead
c
OP50 pellet
HT115 pellet
OP50 pellet
HT115 pellet
+LB
+LB
+HTS
+OPS
d
ns
ns
****
****
number of aggregates
100
80
60
40
20
0
OP50
HT115
OP50
HT115
+LB
+HTS +OPS

Supplementary Figure 4. Bacterial content and not bacteria-host live interactions promotes proteostasis in *C. elegans*. (a) Representative images of 2-day old polyQ40::YFP-expressing worms on alive or UV-killed bacterial lawns. (b) Quantification of polyQ40::YFP fluorescent foci of 2-day old worm cultured on alive or UV-killed bacterial lawns. The number of aggregates per worm is shown. Number of worms, *n* = 36 (alive OP50), *n* = 25 (alive HT115), *n* = 30 (dead OP50), *n* = 26 (dead HT115). OP50 vs. HT115 (alive) $P < 0.0001$, OP50 vs. HT115 (dead) $P < 0.0001$, OP50 (alive) vs. OP50 (dead) $P = 0.346$, HT115 (alive) vs. HT115 (dead) $P = 0.9569$. (c) Representative images of 2-day old polyQ40::YFP-expressing worms on OP50 or HT115 bacterial pellets supplemented with LB media (+LB), supernatants from HT115 cultures (+HTS) or supernatants from OP50 bacterial cultures (+OPS). (d) Quantification of polyQ40::YFP fluorescent foci of 2-day old worm cultured on bacterial pellets supplemented with LB media (+LB), supernatants from HT115 cultures (+HTS) or supernatants from OP50 bacterial cultures (+OPS). The number of aggregates per worm is shown. Number of worms, *n* = 47 (OP50 pellet +LB), *n* = 52 (HT115 pellet +LB), *n* = 34 (OP50 pellet +HTS), *n* = 55 (OP50 pellet +OPS). OP50 vs. HT115 (+LB) $P < 0.0001$, OP50 (+LB) vs. OP50 (+HTS) $P = 0.1747$, HT115 (+LB) vs. HT115 (+OPS) $P = 0.1458$, OP50 (+HTS) vs. HT115 (+OPS) $P < 0.0001$. Values represent mean ± SD from three independent experiments. One-way ANOVA with Sidak's multiple comparison test was used. ns $P>0.05$, ****$P<0.0001$. Scale bars in all panels are 100μm. Source data are provided as a Source Data file.

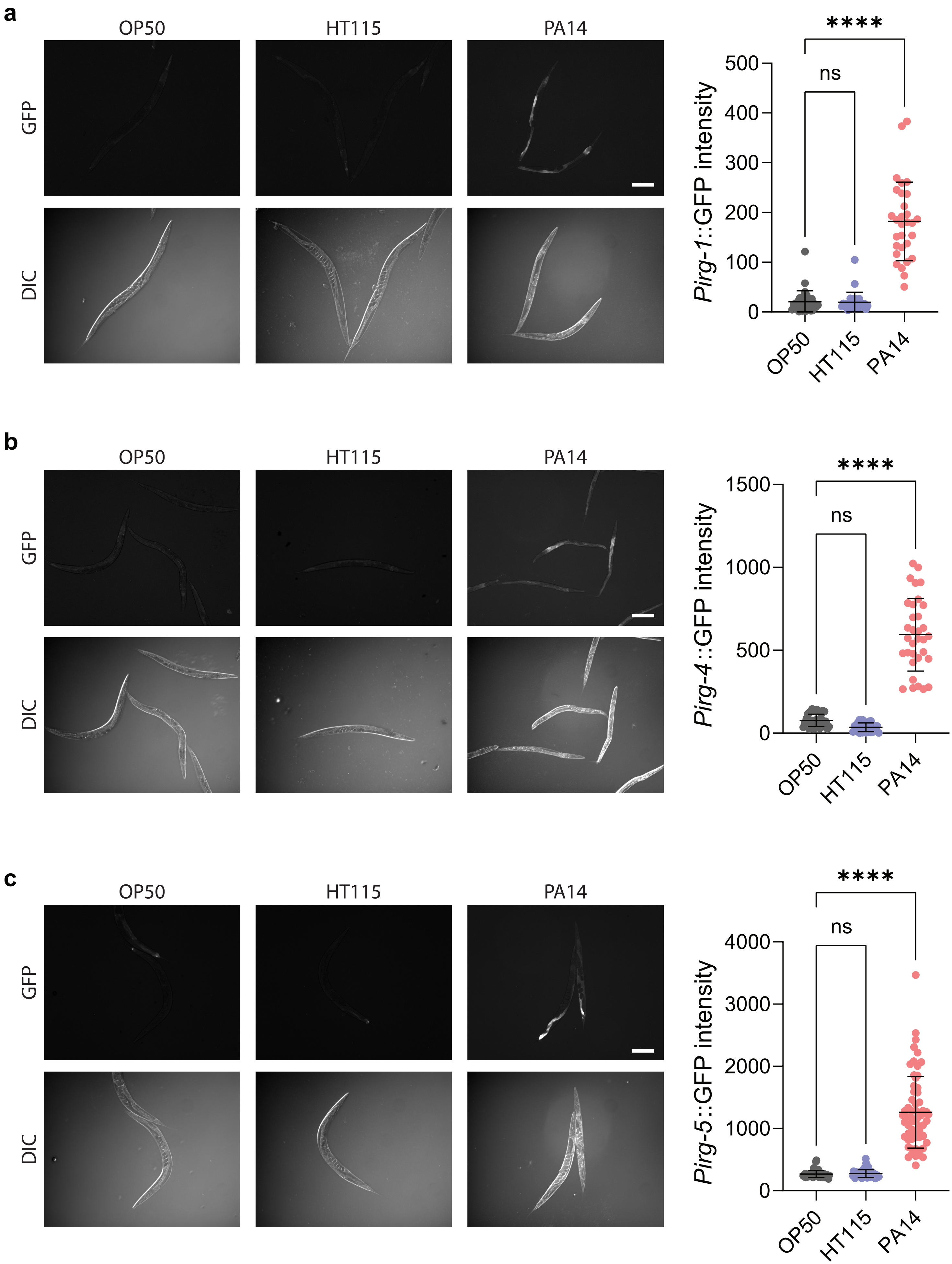

a
OP50
HT115
PA14
GFP
DIC
****
ns
500
400
300
200
100
0
Pirg-1::GFP intensity
OP50
HT115
PA14
b
OP50
HT115
PA14
GFP
DIC
****
ns
1500
1000
500
0
Pirg-4::GFP intensity
OP50
HT115
PA14
c
OP50
HT115
PA14
GFP
DIC
****
ns
4000
3000
2000
1000
0
Pirg-5::GFP intensity
OP50
HT115
PA14

Supplementary Figure 5. The expression levels of *irg* genes are not affected by OP50 and HT115 bacteria. (a-c) Representative fluorescence (upper panel) and DIC (lower panel) images of p_{irg-1}::GFP (a), p_{irg-4}::GFP (b) and p_{irg-5}::GFP (c) expressing worms on OP50, HT115 and *P. aeruginosa* (PA14) bacteria. Quantification of the mean fluorescence intensity is shown on the right. Number of worms, *n* = 32 (OP50), *n* = 25 (HT115), *n* = 30 (PA14) and OP50 vs. HT115 P = 0.9985, OP50 vs. PA14 P < 0.0001 for (a), *n* = 44 (OP50), *n* = 28 (HT115), *n* = 33 (PA14) and OP50 vs. HT115 P = 0.3055, OP50 vs. PA14 P < 0.0001 for (b), *n* = 55 (OP50), *n* = 60 (HT115), *n* = 64 (PA14) and OP50 vs. HT115 P = 0.9892, OP50 vs. PA14 P < 0.0001 for (c). Values are in arbitrary units and represent mean ± SD. One-way ANOVA with Sidak's multiple comparison test was used. ns P>0.05, **** P<0.0001. Scale bars is 200μm. Source data are provided as a Source Data file.

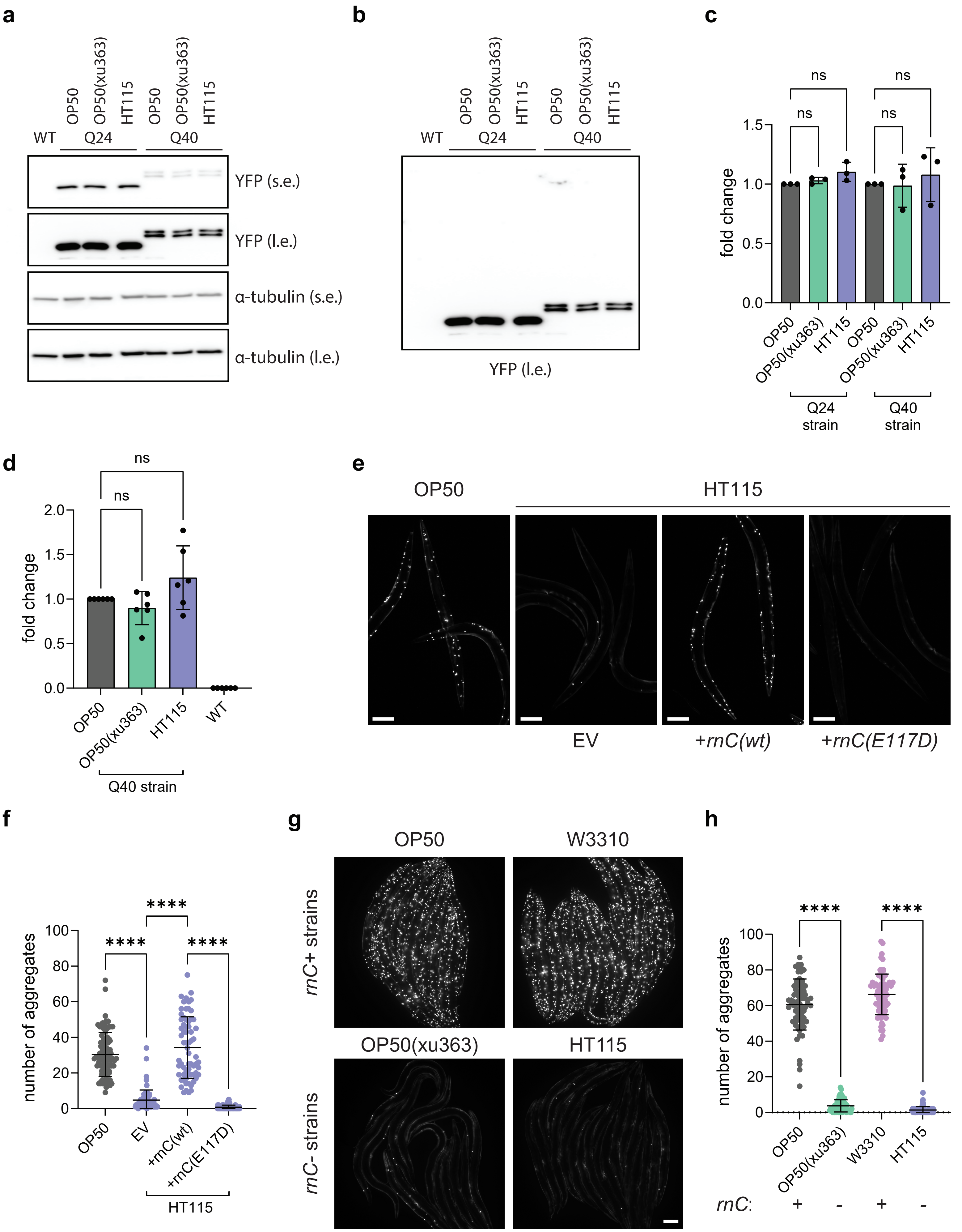

a
WT
Q24
OP50
OP50(xu363)
HT115
Q40
OP50
OP50(xu363)
HT115
YFP (s.e.)
YFP (l.e.)
α-tubulin (s.e.)
α-tubulin (l.e.)
b
WT
Q24
OP50
OP50(xu363)
HT115
Q40
OP50
OP50(xu363)
HT115
YFP (l.e.)
c
fold change
ns
ns
ns
ns
1.5
1.0
0.5
0.0
OP50
OP50(xu363)
HT115
OP50
OP50(xu363)
HT115
Q24 strain
Q40 strain
d
fold change
ns
ns
2.0
1.5
1.0
0.5
0.0
OP50
OP50(xu363)
HT115
WT
Q40 strain
e
OP50
HT115
EV
+rnC(wt)
+rnC(E117D)
f
number of aggregates
****
****
****
100
80
60
40
20
0
OP50
EV
+rnC(wt)
+rnC(E117D)
HT115
g
OP50
W3310
rnC+ strains
OP50(xu363)
HT115
rnC- strains
h
number of aggregates
****
****
100
80
60
40
20
0
OP50
OP50(xu363)
W3310
HT115
rnC:
+
-
+
-

Supplementary Figure 6. Ribonuclease 3 expression in bacteria regulates the accumulation of polyQ40 protein aggregates in *C. elegans* but not the levels of polyQ40. (a, b) Representative immunoblots of lysates from whole polyQ24::YFP-expressing (Q24) and polyQ40::YFP-expressing (Q40) 2-day old worms reared on the different diets, probed for GFP and α-tubulin as internal loading control. Lysates for 2-day old WT worms on OP50 were used as negative control. The experiment was repeated three times with similar results. (c) Fold change analysis of polyQ24::YFP (Q24) and polyQ40::YFP (Q40) intensities relative to respective OP50 and normalized to α-tubulin. Three independent experiments were performed. OP50 vs. OP50(xu363) (Q24) P = 0.9972, OP50 vs. HT115 (Q24) P = 0.7925, OP50 vs. OP50(xu363) (Q40) P = 0.9999, OP50 vs. HT115 (Q40) P = 0.9033. (d) Fold change RT-PCR analysis of YFP::GFP from polyQ40::YFP (Q40) worms on OP50, OP50(xu363) and HT115 diets relative to OP50. WT animals were used as negative control. Three independent experiments that were run twice, were performed. OP50 vs. OP50(xu363) P = 0.7800, OP50 vs. HT115 P = 0.1480. (e) Representative images of 2-day old polyQ40::YFP-expressing worms on OP50 and HT115 expressing the wt (+*rnC(wt)*) or catalytically dead (+*rnC(E117D)*) ribonuclease 3. EV serves as the control vector. Scale bar is 100μm. (f) Quantification of polyQ40::YFP fluorescent foci of 2-day old worms on OP50 and HT115 bacteria expressing the control empty vector (EV), wt (+*rnC(wt)*) or catalytically dead (+*rnc(E117K)*) ribonuclease 3. Number of worms, *n* = 89 (OP50), *n* = 74 (EV), *n* = 67 (+*rnC(wt)*), *n* = 65 (+*rnC(E117K)*), *n* = 75 (+*rnC(E117D)*). OP50 vs. EV P < 0.0001, EV vs. +*rnc(wt)* P < 0.0001, EV vs. +*rnC(E117D)* P = 0.1004, +*rnC(wt)* vs. +r*nC(E117D)* P < 0.0001. (g) Representative images of 2-day old polyQ40::YFP-expressing worms on ribonuclease 3 expressing (*rnC*+) OP50 and W3310 paternal *E. coli* or their ribonuclease depleted (*rnC-)* OP50(xu363) and HT115 derivatives. Scale bar is 100μm. (h) Quantification of polyQ40::YFP fluorescent foci of 2-day old worms on ribonuclease expressing (*rnC*+) OP50 and W3310 *E. coli* or ribonuclease depleted (*rnC*-) OP50(xu363) and HT115 bacteria. The number of aggregates per worm is shown. Number of worms, *n* = 70 (OP50), *n* = 62 (OP50(*rnc*-)), *n* = 72 (W3310) *n* = 78 (HT115(*rnC*-)). OP50 vs. OP5*0(xu363)* P < 0.0001*,* OP50 vs. W3310 P = 0.0011, OP50(xu363) vs. W3310 P < 0.0001. Values represent mean ± SD from three independent experiments. One-way ANOVA with Sidak's multiple comparison test was used. ns P>0.05, **** P<0.0001. Source data are provided as a Source Data file.

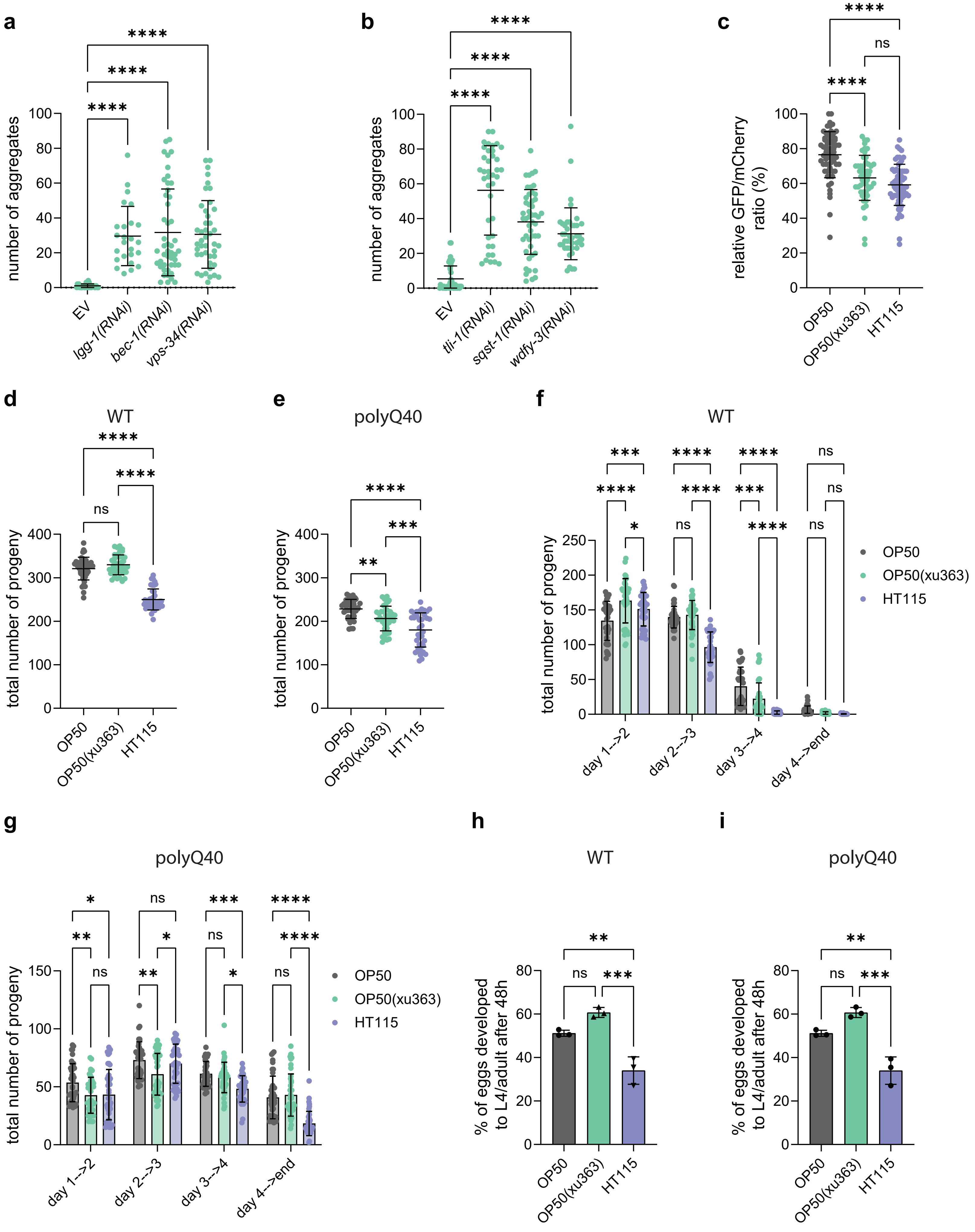
a
number of aggregates
EV
lgg-1(RNAi)
bec-1(RNAi)
vps-34(RNAi)
b
number of aggregates
EV
tli-1(RNAi)
sqst-1(RNAi)
wdfy-3(RNAi)
c
relative GFP/mCherry ratio (%)
OP50
OP50(xu363)
HT115
d
WT
total number of progeny
e
polyQ40
total number of progeny
f
WT
total number of progeny
day 1-->2
day 2-->3
day 3-->4
day 4-->end
OP50
OP50(xu363)
HT115
g
polyQ40
total number of progeny
h
WT
% of eggs developed to L4/adult after 48h
i
polyQ40
% of eggs developed to L4/adult after 48h

Supplementary Figure 7. Autophagy disruption increases protein aggregation in worms on OP50(xu363) diet and lesion of ribonuclease 3 in OP50 bacteria (OP50(xu363)) does not negatively affect *C. elegans* brood size or developmental rate. (a) Quantification of polyQ40::YFP fluorescent foci of 2-day old worms on OP50(xu363), treated with EV, *lgg-1(RNAi), bec-1(RNAi)* and *vps-34(RNAi)*. The number of aggregates per worm is shown. Number of worms, *n* = 37 (EV), *n* = 25 (*lgg-1(RNAi)*), *n* = 37 (*bec-1(RNAi)*), *n* = 43 (*vps-34(RNAi)*). P > 0.0001. (b) Quantification of polyQ40::YFP fluorescent foci of 2-day old worms on OP50(xu363), treated with EV, *tli-1(RNAi), sqst-1(RNAi)* and *wdfy-3(RNAi)*. The number of aggregates per worm is shown. Number of worms, *n* = 42 (EV), *n* = 39 (*tli-1(RNAi)*), *n* = 48 (sqst-*1(RNAi)*), *n* = 41 (*wdfy-3(RNAi)*). P > 0.0001. (c) Quantification of GFP/mCherry LGG-1 positive puncta per muscle cell of worms on OP50, OP50(xu363) and HT115. n = 64 (OP50), n = 52 (OP50(xu363)), n = 63 (HT115). OP50 vs. OP50(xu363) P < 0.0001, OP50 vs. HT115 P < 0.0001, OP50(xu363) vs. HT115 P = 0.2582. (d) Total brood size and (f) brood size per day of wt worms on OP50, OP50(xu363) and HT115. (e) Total brood size and (g) brood size per day of polyQ40expressing worms on OP50, OP50(xu363) and HT115. Number of worms, *n* = 40 for all conditions (d-g). (d) OP50 vs. OP50(xu363) P = 0.2977, OP50 vs. HT115 P < 0.0001, OP50(xu363) vs. HT115 P < 0.0001. (e) OP50 vs. OP50(xu363) P = 0.0048, OP50 vs. HT115 P < 0.0001, OP50(xu363) vs. HT115 P = 0.0007. (f) day 1-->2 OP50 vs. OP50(xu363) P < 0.0001, OP50 vs. HT115 P = 0.0007, OP50(xu363) vs. HT115 P = 0.0196, day 2-->3 OP50 vs. OP50(xu363) P = 0.7676, OP50 vs. HT115 P < 0.0001, OP50(xu363) vs. HT115 P < 0.0001, day 3-->4 OP50 vs. OP50(xu363) P = 0.0002, OP50 vs. HT115 P < 0.0001, OP50(xu363) vs. HT115 P < 0.0001, day 4-->end OP50 vs. OP50(xu363) P = 0.5123, OP50 vs. HT115 P = 0.3344, OP50(xu363) vs. HT115 P = 0.09482. (g) day 1-->2 OP50 vs. OP50(xu363) P = 0.0069, OP50 vs. HT115 P = 0.0109, OP50(xu363) vs. HT115 P = 09880, day 2-->3 OP50 vs. OP50(xu363) P = 0.0020, OP50 vs. HT115 P = 0.6758, OP50(xu363) vs. HT115 P = 0.0279, day 3-->4 OP50 vs. OP50(xu363) P = 0.6699, OP50 vs. HT115 P = 0.0009, OP50(xu363) vs. HT115 P = 0.0153, day 4-->end OP50 vs. OP50(xu363) P = 0.8326, OP50 vs. HT115 P < 0.0001, OP50(xu363) vs. HT115 P < 0.0001.

(h) Developmental rate of wt and (i) polyQ40-expressing worms on OP50, OP50(xu363) and HT115, was measured as the percentage of eggs that developed into L4/adult stages 48h and 62h after egg laying, respectively. Number of worms, n = 751 (OP50), n = 870 (OP50(xu363)), n = 619 (HT115) for (h), n = 883 (OP50), n = 875 (OP50(xu363)), n = 1030 (HT115) for (i). (h) OP50 vs. OP50(xu363) P = 0.0732, OP50 vs. HT115 P = 0.0053, OP50(xu363) vs. HT115 P = 0.0005. (i) OP50 vs. OP50(xu363) P = 0.9107, OP50 vs. HT115 P = 0.015, OP50(xu363) vs. HT115 P = 0.0007.Values represent mean ± SD from three independent experiments. One-way ANOVA (a-e, h, i) with Sidak's multiple comparison test and 2way ANOVA (f, g) with Tukey's multiple comparison test were used. ns P>0.05, * P<0.05, ** P<0.01, *** P<0.001, **** P<0.0001. Source data are provided as a Source Data file.

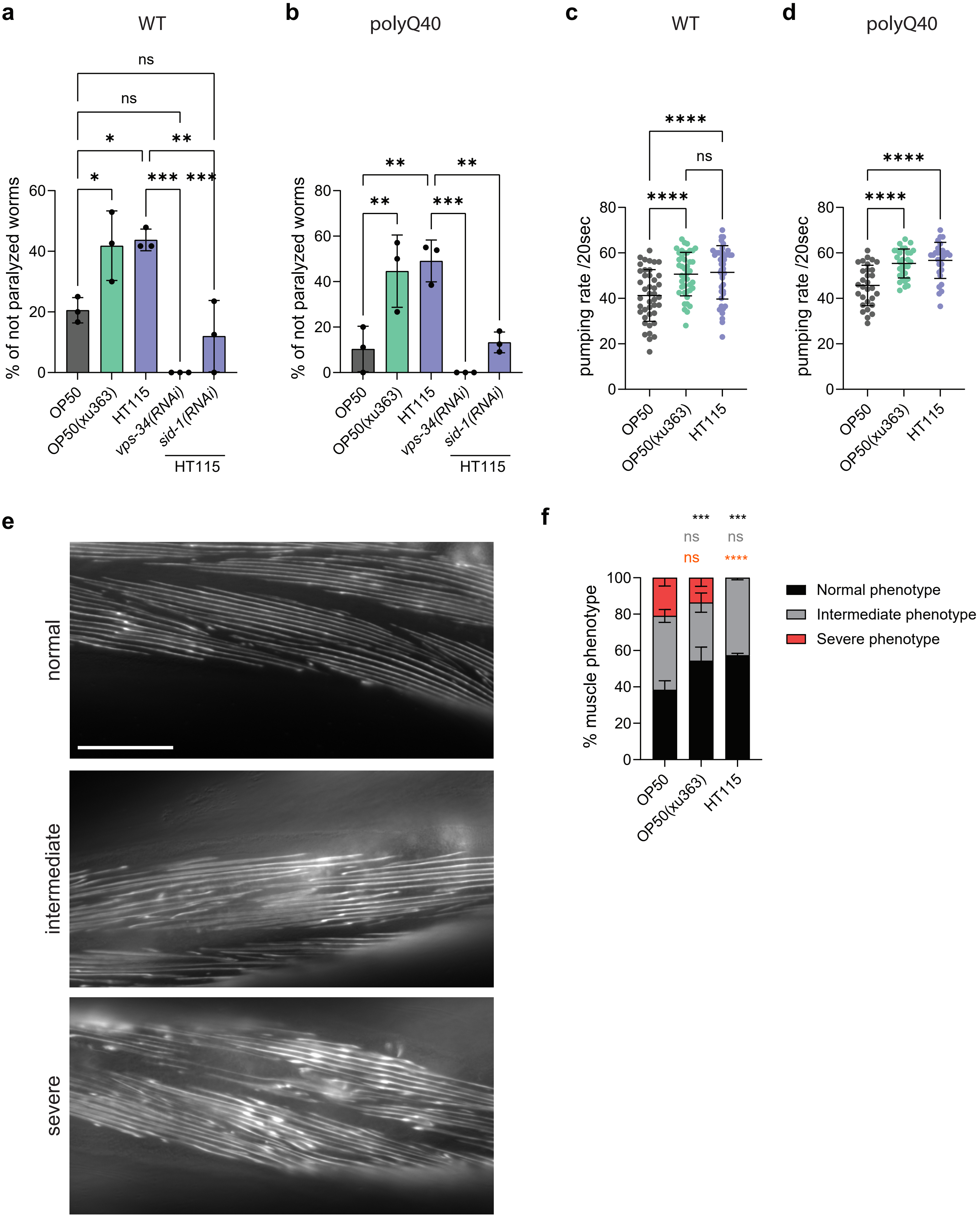

a
WT
ns
ns
*
**
*
***
***
% of not paralyzed worms
60
40
20
0
OP50
OP50(xu363)
HT115
vps-34(RNAi)
sid-1(RNAi)
HT115
b
polyQ40
**
**
**
***
% of not paralyzed worms
80
60
40
20
0
OP50
OP50(xu363)
HT115
vps-34(RNAi)
sid-1(RNAi)
HT115
c
WT
****
ns
****
pumping rate /20sec
80
60
40
20
0
OP50
OP50(xu363)
HT115
d
polyQ40
****
****
pumping rate /20sec
80
60
40
20
0
OP50
OP50(xu363)
HT115
e
normal
intermediate
severe
f
***
***
ns
ns
ns
****
% muscle phenotype
100
80
60
40
20
0
OP50
OP50(xu363)
HT115
Normal phenotype
Intermediate phenotype
Severe phenotype

Supplementary Figure 8. Lesion of ribonuclease 3 in OP50 bacteria (OP50(xu363)) positively affects motility, pharyngeal pumping and muscle phenotype. (a) Percentage of not paralyzed (18-20 days old) wt worms on different bacterial diets was assessed. Number of worms *n* = 67 (OP50), *n* = 61 (OP50(xu363)) *n* = 48 (HT115), *n* = 23 (*vps-34(RNAi)*), *n* = 35 (*sid-1(RNAi)*). OP50 vs. OP50(xu363) P = 0.0425, OP50 vs. HT115 P = 0.0257, OP50 vs. *vps-34(RNAi)* P = 0.0511, OP50 vs *sid-1(RNAi)* P = 0.7484, HT115 vs. *vps-34(RNAi)* P = 0.0002, , HT115 vs. *sid-1(RNAi)* P = 0.0031 (b) Percentage of not paralyzed (16-18 days old) polyQ40-expressing worms on different bacterial diets was assessed. Number of worms *n* = 49 (OP50), *n* = 52 (OP50(xu363)) *n* = 46 (HT115), *n* = 18 (*vps-34(RNAi)*), *n* = 30 (*sid-1(RNAi)*). OP50 vs. OP50(xu363) P = 0.0055, OP50 vs. HT115 P = 0.0023, HT115 vs. *vps-34(RNAi)* P = 0.0004, , HT115 vs. *sid-1(RNAi)* P = 0.0040. Pumping rate per 20 seconds of 8-day old (c) wt and (d) polyQ40-expressing worms was assed. Number of worms for (c) *n* = 42 (OP50), *n* = 42 (OP50(xu363)) *n* = 42 (HT115) and for (d) *n* = 30 (OP50), *n* = 30 (OP50(xu363)) *n* = 30 (HT115). (c) OP50 vs. OP50(xu363) P < 0.0001, OP50 vs. HT115 P < 0.0001, OP50(xu363) vs. HT115 P = 0.1102. (d) All P < 0.0001. (e) Muscles were classified into three categories depending on their morphology (normal, intermediated and severe phenotype). Representative fluorescence images of 8-day old GFP::myo-3-expressing worms of each category are shown. Scale Bar is 20µm. (f) Quantification of the percentage of muscle phenotype of 8-day old GFP::myo-3-expressing worms grown on OP50, OP50(xu363) or HT115. Number of muscles analysed, *n* = 135 (OP50), *n* = 116 (OP50(xu363)), *n* = 151 (HT115). Comparisons are made versus OP50. For normal phenotype, OP50 vs. OP50(xu363) P = 0.0007, OP50 vs. HT115 P = 0.0001, OP50(xu363) vs. HT115 P = 0.6774, for intermediate phenotype, OP50 vs. OP50(xu363) P = 0.0603, OP50 vs. HT115 P = 0.8392, OP50(xu363) vs. HT115 P = 0.0190, for severe phenotype, OP50 vs. OP50(xu363) P = 0.1225, OP50 vs. HT115 P < 0.0001, OP50(xu363) vs. HT115 P = 0.0030. Values represent mean ± SD from at least three independent experiments. One-way ANOVA (a, b) with Sidak's multiple comparison test and 2way ANOVA (c, d, f) with Tukey's multiple comparison test were used. ns P>0.05, * P<0.05, ** P<0.01, *** P<0.001, **** P<0.0001. Source data are provided as a Source Data file.

a

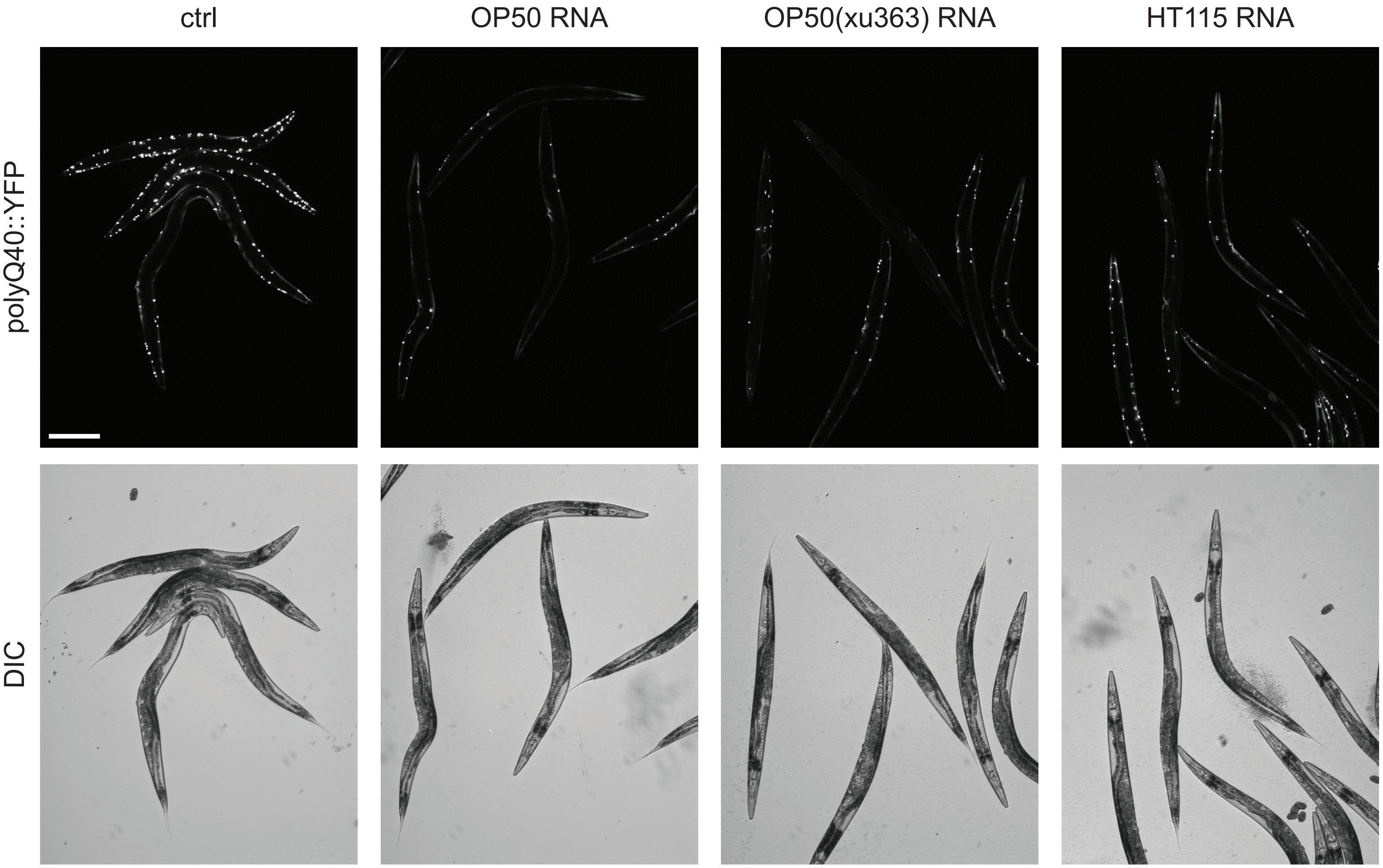

ctrl
OP50 RNA
OP50(xu363) RNA
HT115 RNA
polyQ40::YFP
DIC

b

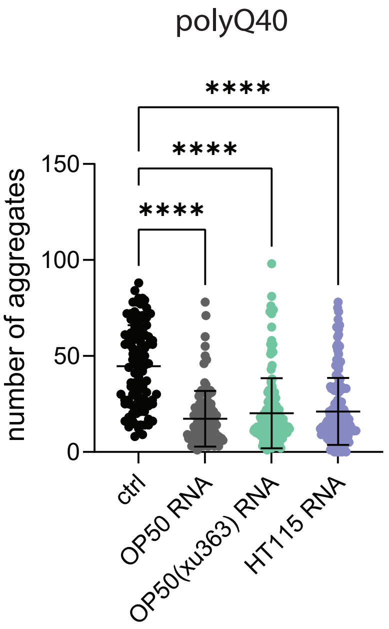

polyQ40
****
****
****
number of aggregates
150
100
50
0
ctrl
OP50 RNA
OP50(xu363) RNA
HT115 RNA

Supplementary Figure 9. Bacterial RNA affects aggregate formation. (a) Representative fluorescence (upper panel) and DIC (lower panel) images of 2-day old poly-Q40::YFP-expressing worms on OP50 diets, descendants of worms in which their gonads were injected with water (ctrl), or RNA derived from OP50, OP50(xu363) or HT115 bacteria. Scale bar is 200μm. (b) Quantification of polyQ40::YFP fluorescent foci of 2-day old worms on OP50 bacterial diets, descendants of worms in which their gonads were injected with water (ctrl), or RNA derived from OP50, OP50(xu363) or HT115 bacteria. The number of aggregates per worm is shown. Number of worms, *n* = 99 (ctrl), *n* = 123 (OP50 RNA), *n* = 143 (OP50(xu363) RNA), *n* = 177 (HT115 RNA). Values represent mean ± SD from three independent experiments. One-way ANOVA with Sidak's multiple comparison test was used. **** $P<0.0001$. Source data are provided as a Source Data file.

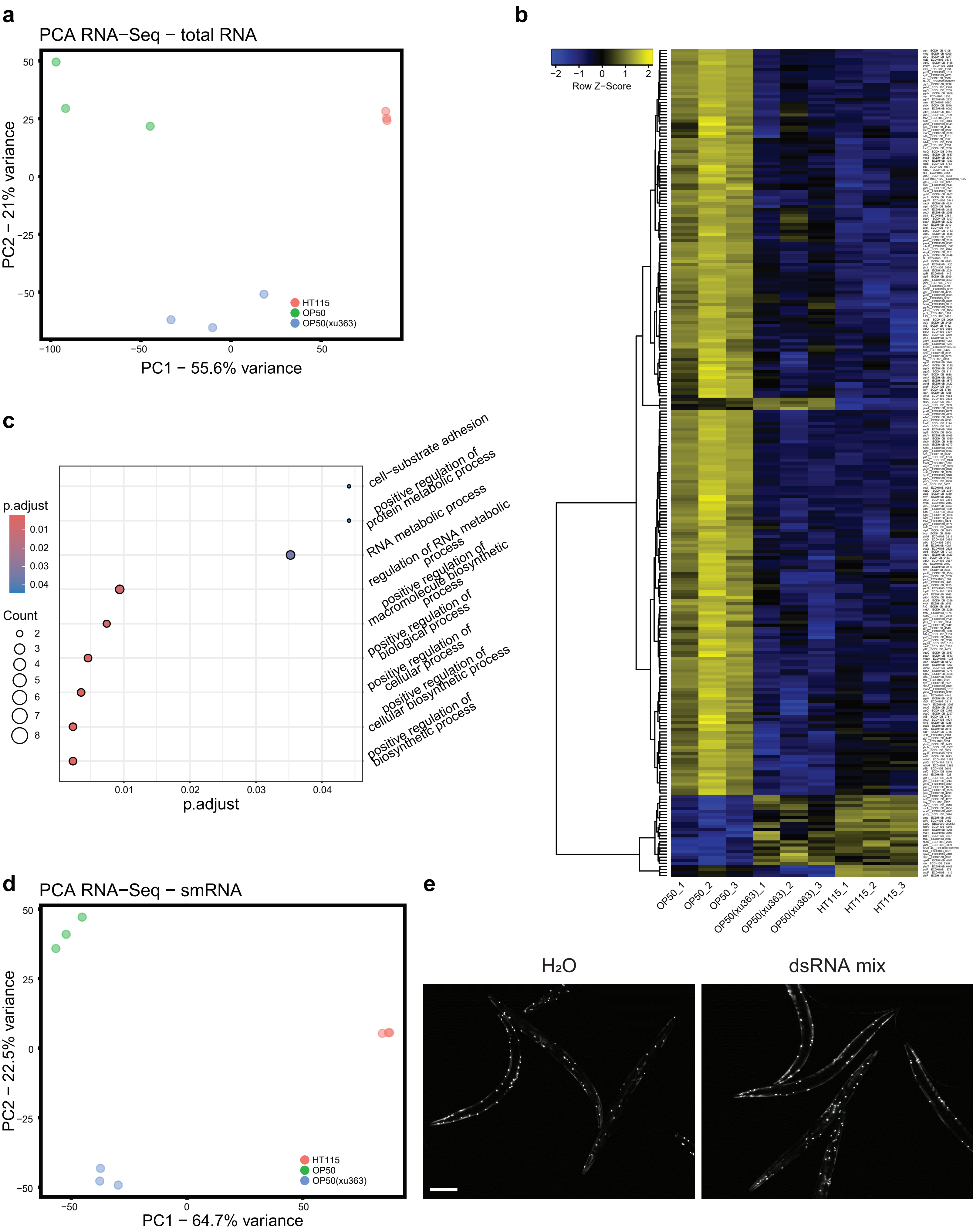

a
PCA RNA−Seq − total RNA
PC2 − 21% variance
PC1 − 55.6% variance
HT115
OP50
OP50(xu363)
b
Row Z−Score
−2 −1 0 1 2
OP50_1
OP50_2
OP50_3
OP50(xu363)_1
OP50(xu363)_2
OP50(xu363)_3
HT115_1
HT115_2
HT115_3
c
p.adjust
Count
cell−substrate adhesion
positive regulation of protein metabolic process
RNA metabolic process
regulation of RNA metabolic process
positive regulation of macromolecule biosynthetic process
positive regulation of biological process
positive regulation of cellular process
positive regulation of cellular biosynthetic process
positive regulation of biosynthetic process
d
PCA RNA−Seq − smRNA
PC2 − 22.5% variance
PC1 − 64.7% variance
HT115
OP50
OP50(xu363)
e
H₂O
dsRNA mix

Supplementary Figure 10. RNA-seq analysis of OP50, OP50(xu363), and HT115 *E. coli* strains. (a) Principal component analysis of total RNA-Seq read counts mapped to genes of the standard *E. coli* K12 genome. Read counts were normalized using the variance stabilizing transformation as implemented in DESeq2. (b) Heatmap showing expression of patterns of differentially expressed genes from the total RNA-Seq experiments. (c) Dotplot showing enriched Gene Ontology biological pathways among genes consistently up-regulated in HT115 and OP50xu363 compared to OP50. (d) Principal component analysis of small RNA-Seq read counts mapped to the collection of *de novo* assembled Trinity contigs. Read counts were normalized using the variance stabilizing transformation as implemented in DESeq2. (e) Representative images of 2-day old poly-Q40::YFP-expressing worms on OP50 diets, descendants of worms in which their gonads were injected with water (H_2O) or dsRNA mix produced by *in vitro* transcription . Scale bar is 200μm.

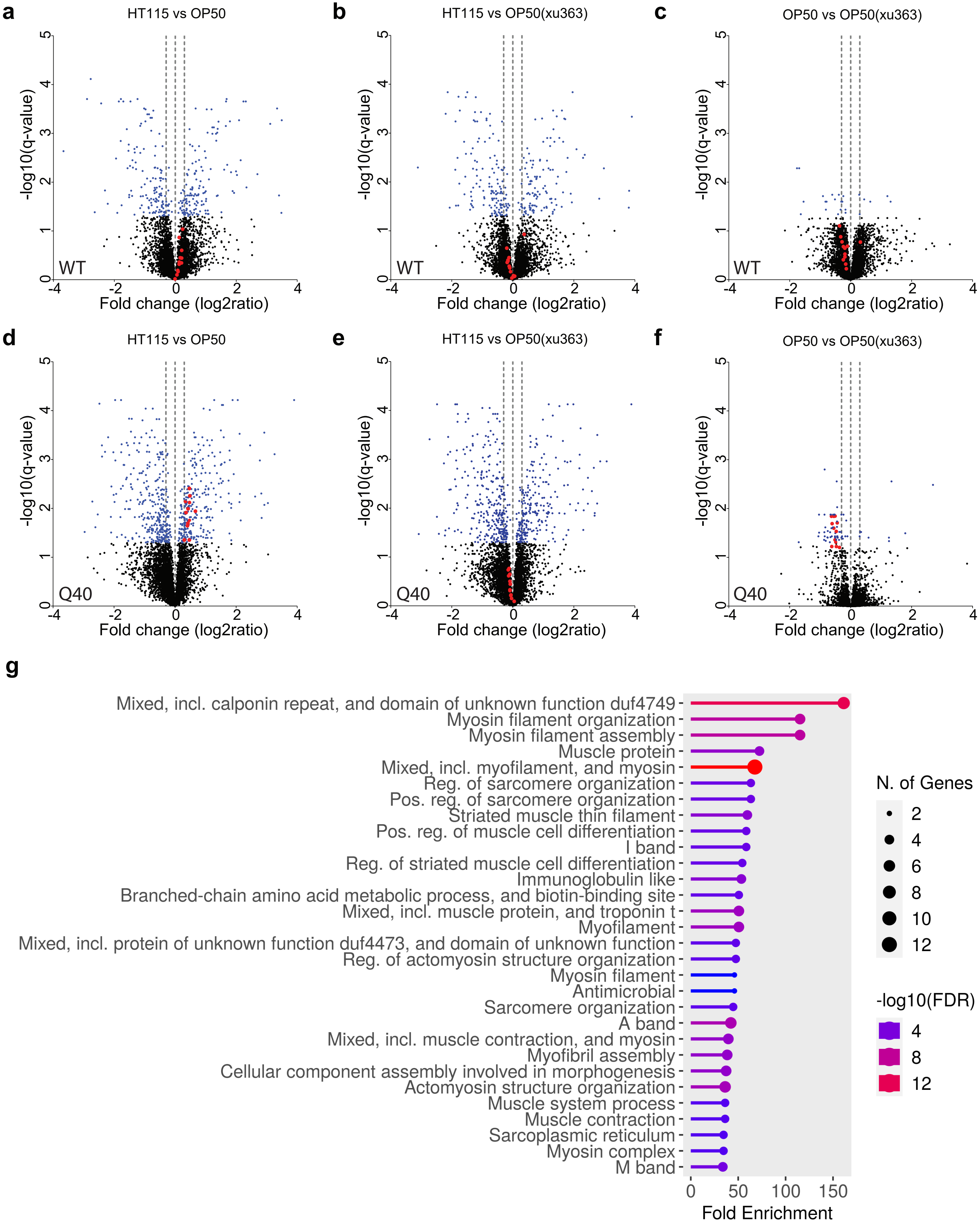

a
HT115 vs OP50
b
HT115 vs OP50(xu363)
c
OP50 vs OP50(xu363)
d
HT115 vs OP50
e
HT115 vs OP50(xu363)
f
OP50 vs OP50(xu363)
-log10(q-value)
Fold change (log2ratio)
WT
Q40
g
Mixed, incl. calponin repeat, and domain of unknown function duf4749
Myosin filament organization
Myosin filament assembly
Muscle protein
Mixed, incl. myofilament, and myosin
Reg. of sarcomere organization
Pos. reg. of sarcomere organization
Striated muscle thin filament
Pos. reg. of muscle cell differentiation
I band
Reg. of striated muscle cell differentiation
Immunoglobulin like
Branched-chain amino acid metabolic process, and biotin-binding site
Mixed, incl. muscle protein, and troponin t
Myofilament
Mixed, incl. protein of unknown function duf4473, and domain of unknown function
Reg. of actomyosin structure organization
Myosin filament
Antimicrobial
Sarcomere organization
A band
Mixed, incl. muscle contraction, and myosin
Myofibril assembly
Cellular component assembly involved in morphogenesis
Actomyosin structure organization
Muscle system process
Muscle contraction
Sarcoplasmic reticulum
Myosin complex
M band
Fold Enrichment
N. of Genes
-log10(FDR)

Supplementary Figure 11. Proteome analysis of polyQ40-expressing worms on OP50, OP50(xu363) or HT115 diet. (a-f) Volcano plots of total quantified proteins showing significant increase or decrease content in WT (a-c) and polyQ40-expressing (d-f) strains on OP50, OP50(xu363) or HT115 bacteria. In blue are the proteins with q values less than 0.05. UNC-89, UNC-22, TTN-1, UNC-15, ATN-1, UNC-54, UNC-87, ZK1321.4, Y43F8B.1, TNT-2, CPN-3, CLIK-1 proteins from the STRING analysis are shown in red. Horizontal dotted lines are at -0.3, 0, +0.3 fold change. (g) GO term enrichment analysis of all 84 proteins that were significantly upregulated in HT115 or OP50(xu363)-fed worms relative to OP50-fed worms is shown. A q-value of less than 0.05 was used to filter significant changes prior to the pathway analyses.

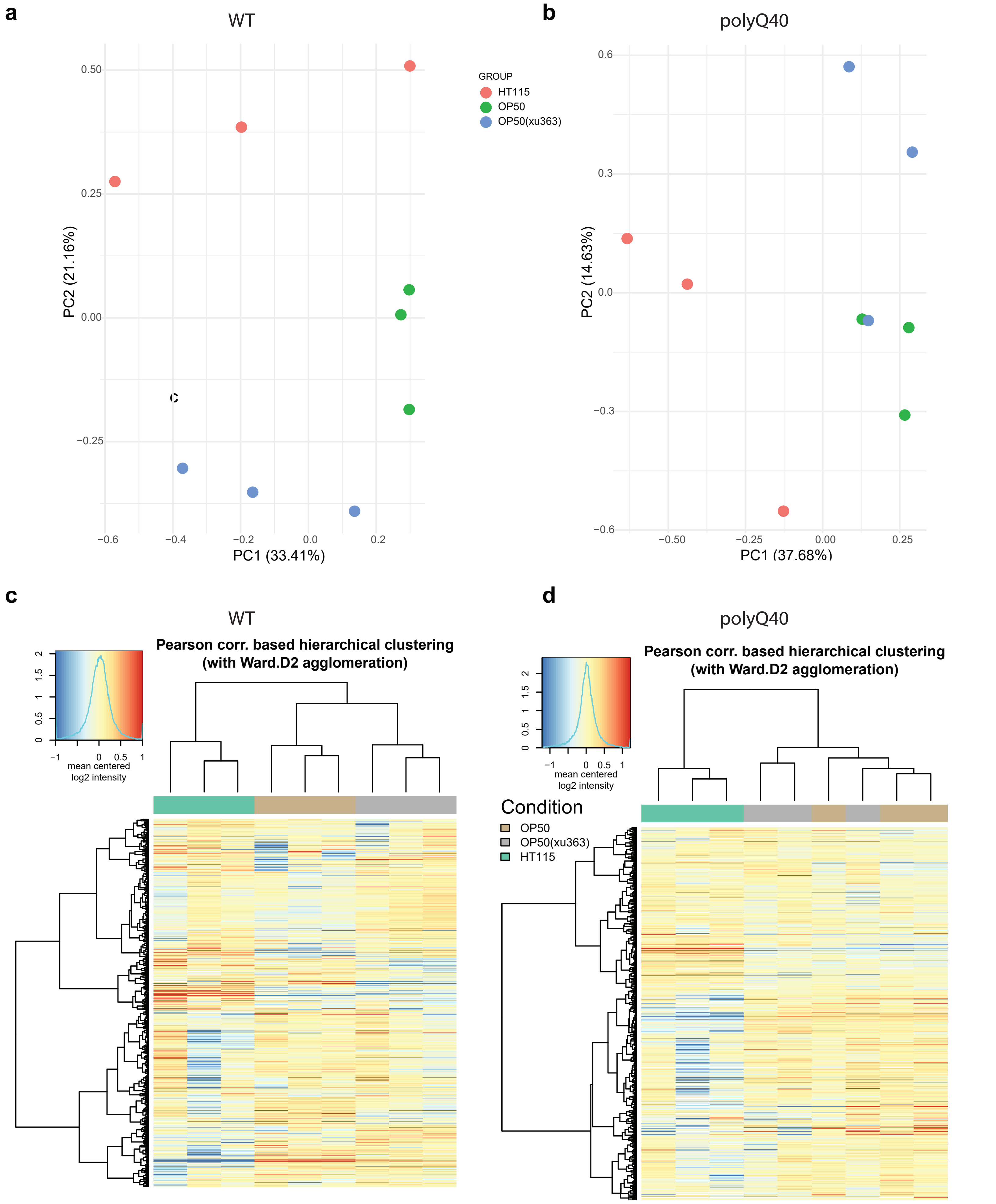
a
WT
PC2 (21.16%)
0.50
0.25
0.00
−0.25
−0.6
−0.4
−0.2
0.0
0.2
PC1 (33.41%)
GROUP
HT115
OP50
OP50(xu363)
b
polyQ40
PC2 (14.63%)
0.6
0.3
0.0
−0.3
−0.6
−0.50
−0.25
0.00
0.25
PC1 (37.68%)
c
WT
Pearson corr. based hierarchical clustering
(with Ward.D2 agglomeration)
mean centered
log2 intensity
d
polyQ40
Pearson corr. based hierarchical clustering
(with Ward.D2 agglomeration)
mean centered
log2 intensity
Condition
OP50
OP50(xu363)
HT115

Supplementary Figure 12. Heatmaps and PCA of proteomic data. PCA (a, b) and heatmaps (c, d) of proteomic data for wt (a, c) and polyQ40-expressing animals (b, d).

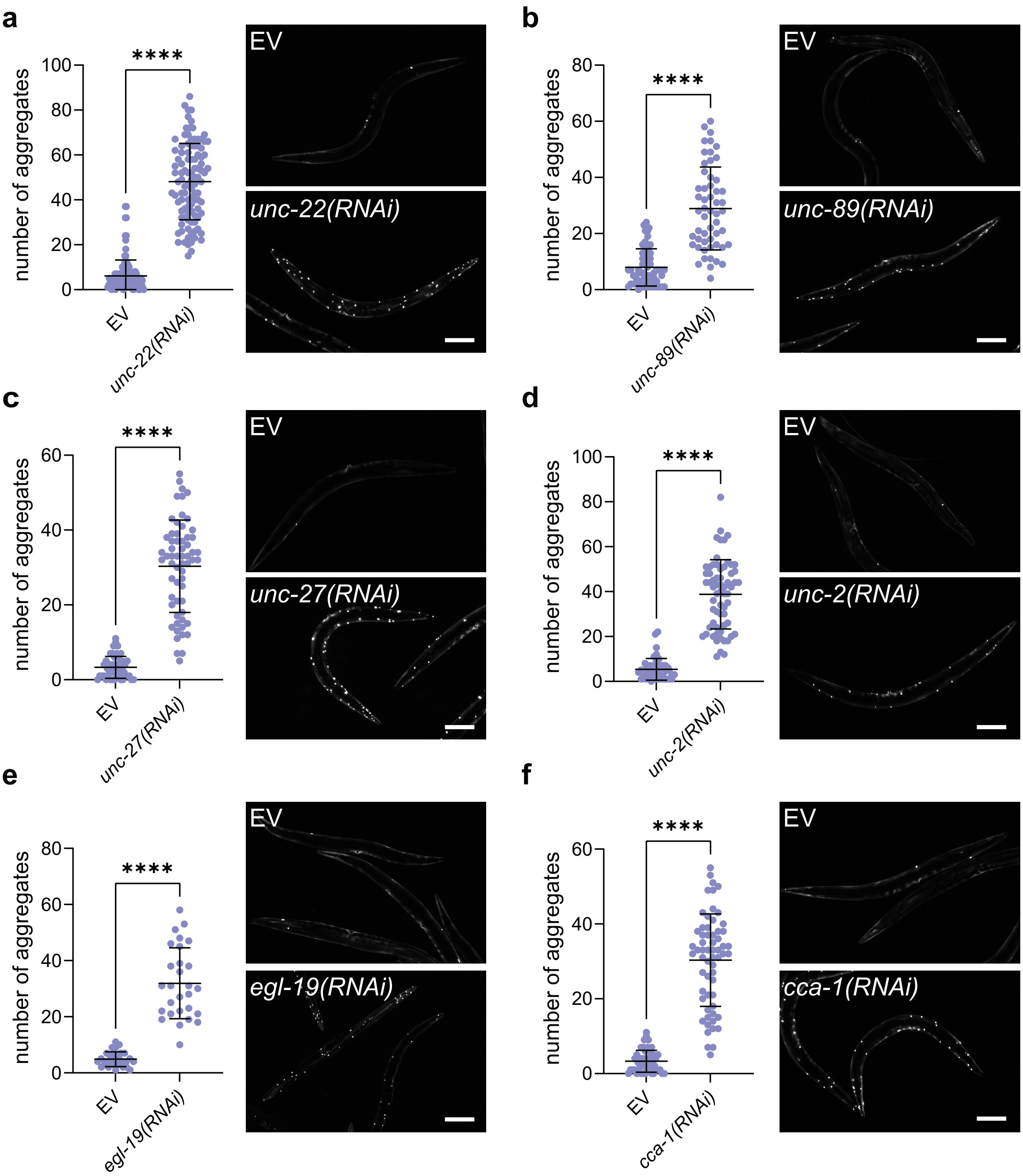

a
b
c
d
e
f
number of aggregates
****
EV
unc-22(RNAi)
unc-89(RNAi)
unc-27(RNAi)
unc-2(RNAi)
egl-19(RNAi)
cca-1(RNAi)

Supplementary Figure 13. Muscle function prevents the accumulation of protein aggregates. (a-f) Quantification of polyQ40::YFP fluorescent foci of 2-day old worms on HT115, treated with empty vector (EV), *unc-22(RNAi)* (a), *unc-89(RNAi)* (b), *unc-27(RNAi)* (c), *unc-2(RNAi)* (d), *egl-19(RNAi)* (e) and *cca-1(RNAi)* (f). The number of aggregates per worm is shown. Number of worms, *n* = 118 (EV), *n* = 104 (*unc-22(RNAi)*) (a), *n* = 63 (EV), *n* = 54 (*unc-89(RNAi)*) (b), *n* = 49 (EV), *n* = 61 (*unc-27(RNAi)*) (c), *n* = 45 (EV), *n* = 64 (*unc-2(RNAi)*) (d), *n* = 30 (EV), *n* = 28 (*egl-19(RNAi)*) (e) *n* = 49 (EV), *n* = 61 (*cca-1(RNAi)*) (f). Representative images are shown on the right of each graph. Values represent mean ± SD from three independent experiments. Mann Whitney t-test was used. **** P<0.0001. Scale bars in all panels are 100µm. Source data are provided as a Source Data file.

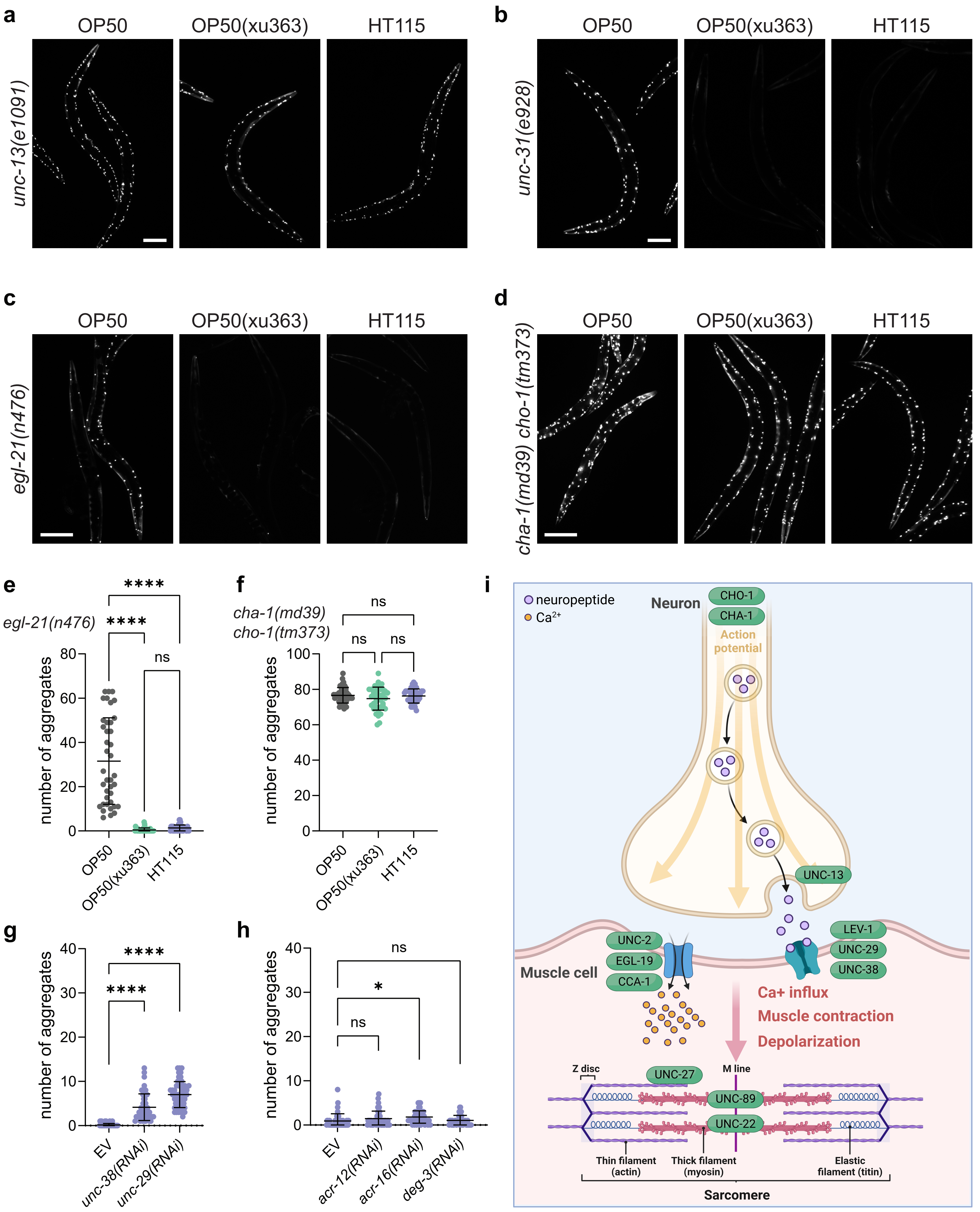

a
OP50
OP50(xu363)
HT115
unc-13(e1091)
b
OP50
OP50(xu363)
HT115
unc-31(e928)
c
OP50
OP50(xu363)
HT115
egl-21(n476)
d
OP50
OP50(xu363)
HT115
cha-1(md39) cho-1(tm373)
e
egl-21(n476)
****
****
ns
number of aggregates
0
20
40
60
80
OP50
OP50(xu363)
HT115
f
cha-1(md39)
cho-1(tm373)
ns
ns
ns
number of aggregates
0
20
40
60
80
100
OP50
OP50(xu363)
HT115
g
****
****
number of aggregates
0
10
20
30
40
EV
unc-38(RNAi)
unc-29(RNAi)
h
ns
*
ns
number of aggregates
0
10
20
30
40
EV
acr-12(RNAi)
acr-16(RNAi)
deg-3(RNAi)
i
neuropeptide
Ca2+
Neuron
CHO-1
CHA-1
Action potential
UNC-13
UNC-2
EGL-19
CCA-1
LEV-1
UNC-29
UNC-38
Muscle cell
Ca+ influx
Muscle contraction
Depolarization
Z disc
UNC-27
M line
UNC-89
UNC-22
Thin filament (actin)
Thick filament (myosin)
Elastic filament (titin)
Sarcomere

Supplementary Figure 14. Acetylcholine signalling protects from polyQ40 aggregation. (a-d) Representative images of 2-day old polyQ40::YFP-expressing worms on OP50, OP50(xu363) and HT115. Images are shown for *unc-13(e1091), unc-31(e928), egl-21(n476)* and *cha-1(md39)cho-1(tm373)* mutant strains. (e, f) Quantification of polyQ40::YFP fluorescent foci of 2-day old worms on OP50, OP50(xu363) and HT115. The number of aggregates per worm is shown in *egl-21(n476)* (e), number of worms, *n* = 39 (OP50), *n* = 37 (OP50(xu363)), *n* = 37 (HT115) and in *cha-1(md39) cho-1(tm373)* (f), number of worms, *n* = 49 (OP50), *n* = 40 (OP50(xu363)), *n* = 44 (HT115), strains. (e) OP50 vs. OP50(xu363) P < 0.0001, OP50 vs. HT115 P < 0.0001, OP50(xu363) vs. HT115 P = 0.9824. (f) OP50 vs. OP50(xu363) P = 0.2177, OP50 vs. HT115 P = 0.9795, OP50(xu363) vs. HT115 P = 0.4180. (g) Quantification of polyQ40::YFP fluorescent foci per 2-day old worms on HT115, treated with empty vector (EV), *unc-38(RNAi)* and *unc-29(RNAi)*. The number of aggregates per worm is shown. Number of worms, *n* = 52 (EV), *n* = 44 (*unc-38(RNAi)*), *n* = 51 (*unc-29(RNAi)*). All P < 0.0001. (h) Quantification of polyQ40::YFP fluorescent foci of 2-day old worms on HT115 diet, treated with EV, *acr-12(RNAi), acr-16(RNAi)* and *deg-3(RNAi)*. The number of aggregates per worm is shown. Number of worms, *n* = 48 (EV), *n* = 46 (*acr-12(RNAi)*), *n* = 46 (*acr-16(RNAi)*), *n* = 45 (*deg-3(RNAi)*). EV vs. *acr-12(RNAi)* P = 0.2231, EV vs. *acr-16(RNAi)* P = 0.0105, EV vs. *deg-3(RNAi)* P = 0.9672. Values represent mean ± SD from three independent experiments. One-way ANOVA with Sidak's multiple comparison test was used. ns P>0.05, * P<0.05, **** P<0.0001. Scale bars in all panels are 200µm. (i) Schematic representation of muscle contraction controlled by calcium influx and acetylcholine receptor activation from neurotransmitter release. Key proteins investigated in this study are shown. Created in BioRender. Spang, A. (2025) https://BioRender.com/s15g98a. Source data are provided as a Source Data file.

a

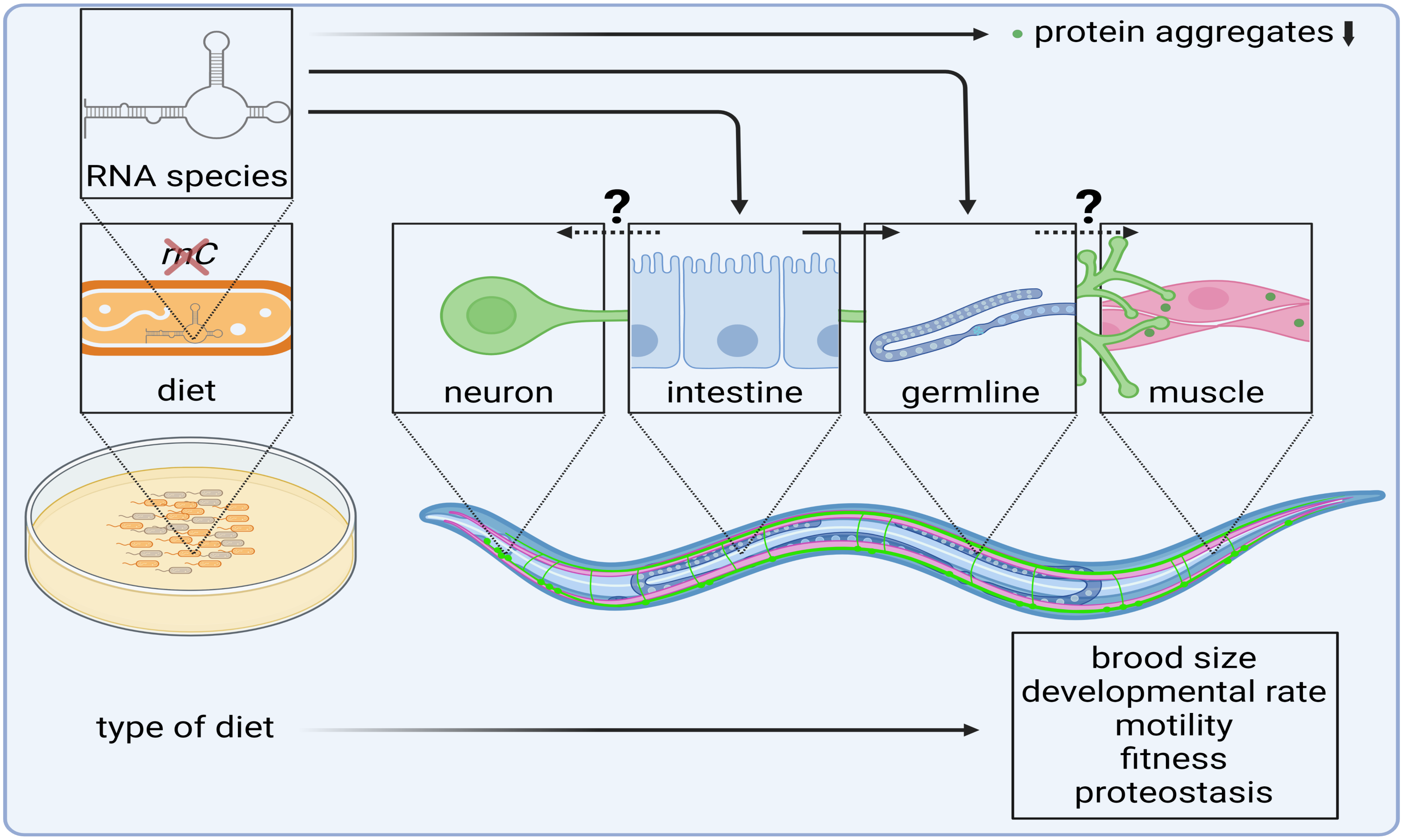
protein aggregates
RNA species
mC
diet
?
?
neuron
intestine
germline
muscle
type of diet
brood size
developmental rate
motility
fitness
proteostasis

Supplementary Figure 15. Schematic representation of our model. Depending on the type of diet the life history traits of *C. elegans* are affected. Bacteria that do not express *rnC* contain RNA species that promote cellular proteostasis and maintain low levels of polyglutamine protein aggregates. The mechanism involved requires communication across tissues. The bacterial-RNA species ingested by the intestinal cells or directly administered in the germline lower the number of aggregates in the muscle cells in a mechanism that requires the RNAi machinery of *C. elegans*. Neuromuscular communication is also required to maintain low levels of protein aggregates. Created in BioRender. Spang, A. (2025) https://BioRender.com/rmcozau.

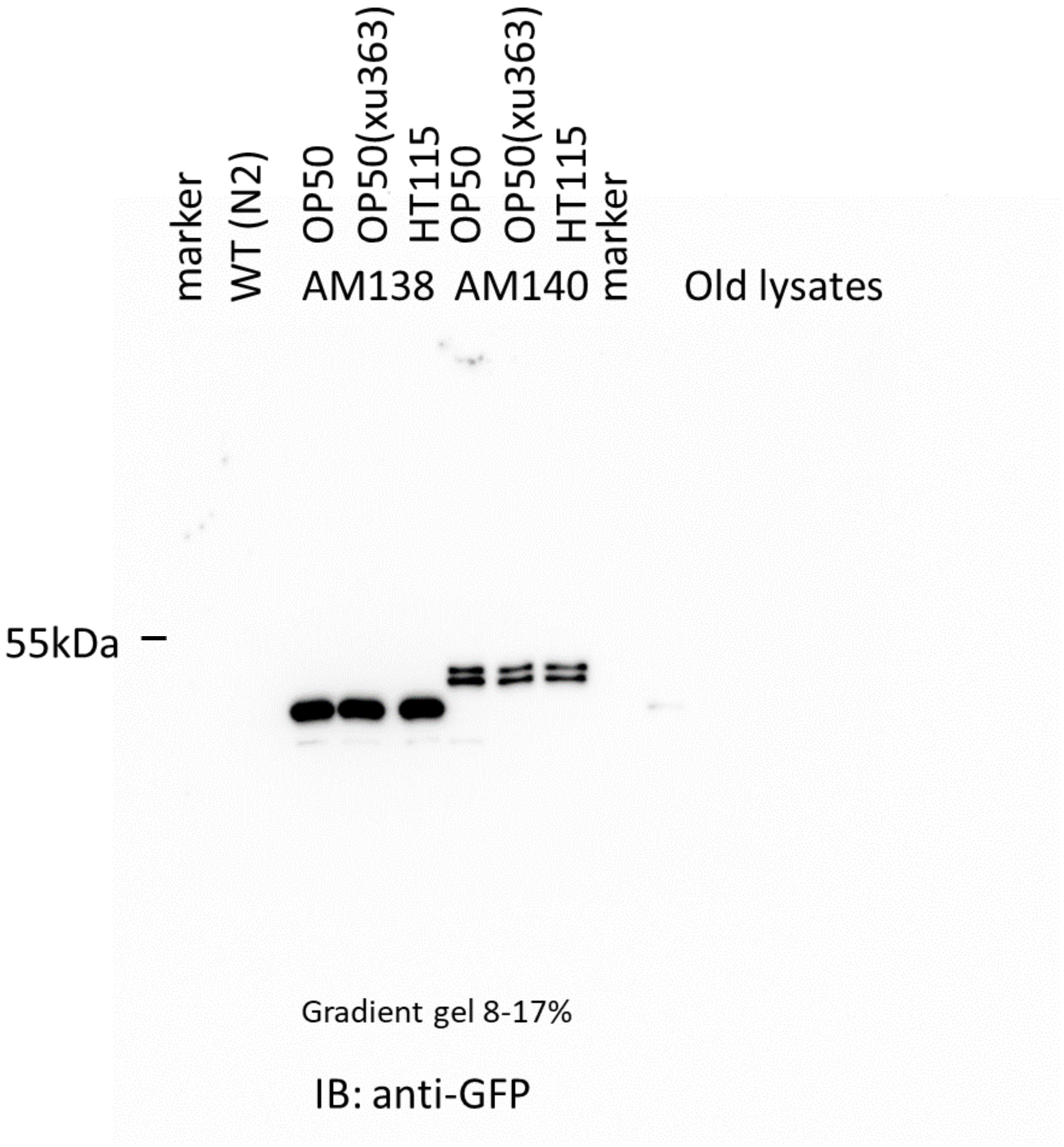
marker
WT (N2)
OP50
OP50(xu363)
HT115
OP50
OP50(xu363)
HT115
marker
AM138
AM140
Old lysates
55kDa
Gradient gel 8-17%
IB: anti-GFP

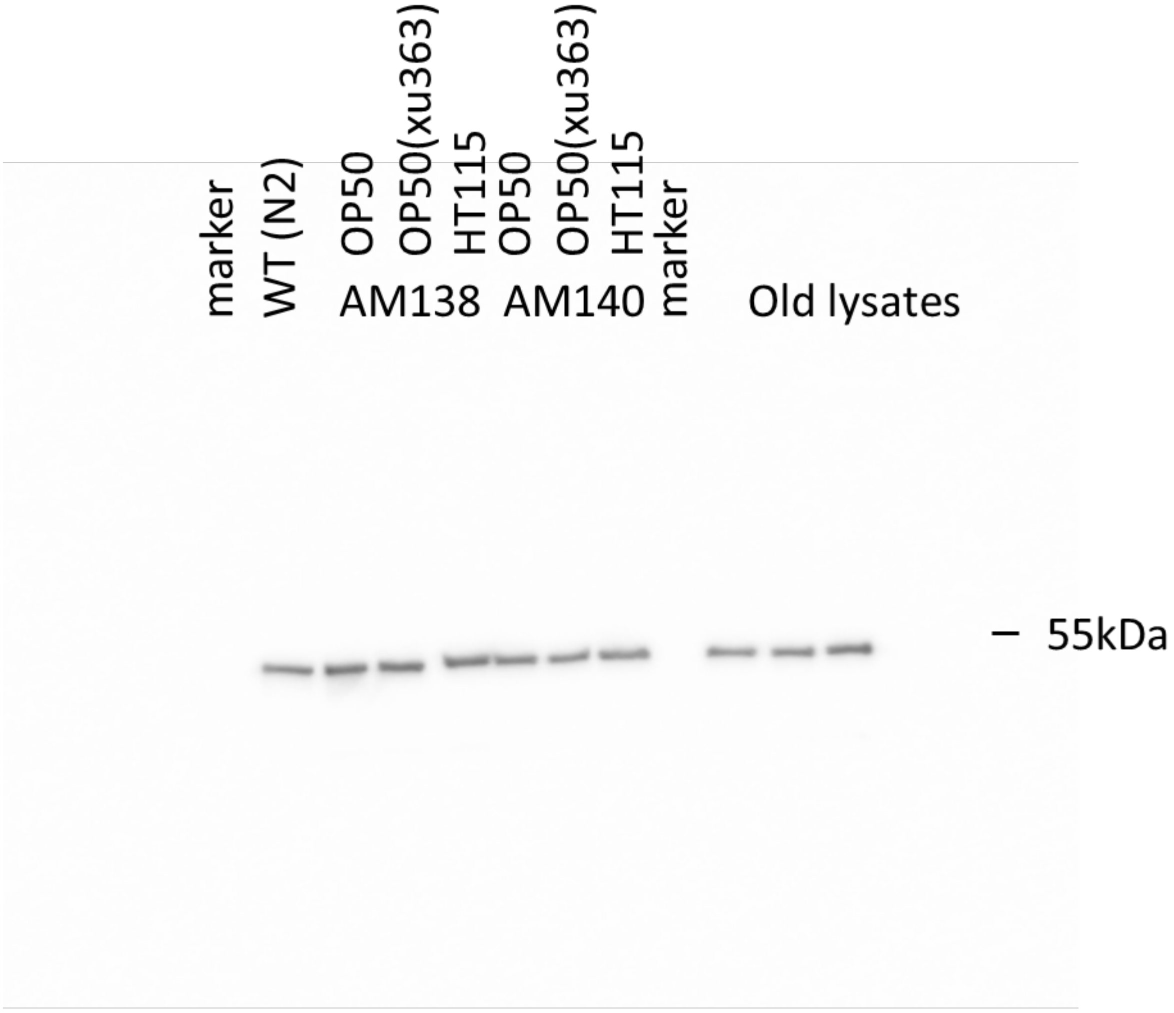

Gradient gel 8-17%

IB: anti-α-tubulin

Uncropped scans of the blots.
