# Supplementary material for: Bacterial RNA promotes proteostasis through inter-tissue communication in C. elegans
Source: Nat Commun. 2025 Oct 1;16:8650. doi: 10.1038/s41467-025-63987-x (PMC12488917; doi:10.1038/s41467-025-63987-x)
Supplement: Supplementary file 2 — Description of Additional Supplementary Files [file 41467_2025_63987_MOESM2_ESM.pdf]

Description of Additional Supplementary Files

File name: Supplementary Movie 1
Description: Wild type worms of advanced age, on OP50 diet.

File name: Supplementary Movie 2
Description: Wild type worms of advanced age, on HT115 diet.

File name: Supplementary Movie 3
Description: Wild type worms of advanced age, on a 1:1 mixture of OP50:HT115 diet.

File name: Supplementary Movie 4
Description: Wild type worms of advanced age, on a 1:10 mixture of OP50:HT115 diet.
