# Supplementary material for: Bacterial RNA promotes proteostasis through inter-tissue communication in C. elegans
Source: Nat Commun. 2025 Oct 1;16:8650. doi: 10.1038/s41467-025-63987-x (PMC12488917; doi:10.1038/s41467-025-63987-x)
Supplement: Supplementary file 7 — Reporting Summary [file 41467_2025_63987_MOESM7_ESM.pdf]

nature portfolio

Corresponding author(s): DARP NCOMMS-24-10471-T

Last updated by author(s): 2025/08/14

Reporting Summary

Nature Portfolio wishes to improve the reproducibility of the work that we publish. This form provides structure for consistency and transparency in reporting. For further information on Nature Portfolio policies, see our Editorial Policies and the Editorial Policy Checklist.

Statistics

For all statistical analyses, confirm that the following items are present in the figure legend, table legend, main text, or Methods section.

n/a	Confirmed	
☐	☒	The exact sample size (*n*) for each experimental group/condition, given as a discrete number and unit of measurement
☐	☒	A statement on whether measurements were taken from distinct samples or whether the same sample was measured repeatedly
☐	☒	The statistical test(s) used AND whether they are one- or two-sided<br>*Only common tests should be described solely by name; describe more complex techniques in the Methods section.*
☒	☐	A description of all covariates tested
☒	☐	A description of any assumptions or corrections, such as tests of normality and adjustment for multiple comparisons
☐	☒	A full description of the statistical parameters including central tendency (e.g. means) or other basic estimates (e.g. regression coefficient) AND variation (e.g. standard deviation) or associated estimates of uncertainty (e.g. confidence intervals)
☐	☒	For null hypothesis testing, the test statistic (e.g. *F*, *t*, *r*) with confidence intervals, effect sizes, degrees of freedom and *P* value noted<br>*Give P values as exact values whenever suitable.*
☒	☐	For Bayesian analysis, information on the choice of priors and Markov chain Monte Carlo settings
☒	☐	For hierarchical and complex designs, identification of the appropriate level for tests and full reporting of outcomes
☒	☐	Estimates of effect sizes (e.g. Cohen's *d*, Pearson's *r*), indicating how they were calculated

Our web collection on statistics for biologists contains articles on many of the points above.

Software and code

Policy information about availability of computer code

Data collection	All images were aquired with Zeiss Axioplan 2 epifluorescence microscope with Axiovision Rel 4.8 or LSM880 point scanning confocal microscope with Zen Blue; For RNAseq NextSeq 500 Illumina System; Fluorometry using the QuantiFluor ONE dsDNA System; Fluorometry using the QuantiFluor ONE dsDNA System; Exploris 480 Mass Spectrometer
Data analysis	For statistical analyses, GraphPad Software v9; MSstats R package v.4.7.3; For RNAseq data analysis, Illumina RTA version 2.11.3; CutAdapt v3.4; STAR v2.7.9; Trimmomatic v0.39; ShinyGO (ver. 0.77); STRING (ver. 12.0); For image analyis, Fiji 2.3; Spectronaut (Biognosys v17.4) for proteomic analysis.

For manuscripts utilizing custom algorithms or software that are central to the research but not yet described in published literature, software must be made available to editors and reviewers. We strongly encourage code deposition in a community repository (e.g. GitHub). See the Nature Portfolio guidelines for submitting code & software for further information.

Data

Policy information about availability of data

All manuscripts must include a data availability statement. This statement should provide the following information, where applicable:

- Accession codes, unique identifiers, or web links for publicly available datasets
- A description of any restrictions on data availability
- For clinical datasets or third party data, please ensure that the statement adheres to our policy

The authors declare that the main data supporting the findings of this study are available within the article and its supplementary information files. Source data are provided with this paper. RNAseq and proteomics data will be made publicly available upon publication. RNAseq data have been uploaded to https://www.ncbi.nlm.nih.gov/geo/query/acc.cgi with accession code GSE261167. Proteomic data have been deposited to the ProteomeXchange Consortium (https://www.proteomexchange.org/) via the MassIVE partner repository (https://massive.ucsd.edu/) with MassIVE data set identifier MSV000094244 and ProteomeXchange identifier PXD050390.

Research involving human participants, their data, or biological material

Policy information about studies with human participants or human data. See also policy information about sex, gender (identity/presentation), and sexual orientation and race, ethnicity and racism.

Reporting on sex and gender	Not applicable
Reporting on race, ethnicity, or other socially relevant groupings	Not applicable
Population characteristics	Not applicable
Recruitment	Not applicable
Ethics oversight	Not applicable

Note that full information on the approval of the study protocol must also be provided in the manuscript.

Field-specific reporting

Please select the one below that is the best fit for your research. If you are not sure, read the appropriate sections before making your selection.

[x] Life sciences   [ ] Behavioural & social sciences   [ ] Ecological, evolutionary & environmental sciences

For a reference copy of the document with all sections, see nature.com/documents/nr-reporting-summary-flat.pdf

Life sciences study design

All studies must disclose on these points even when the disclosure is negative.

Sample size	Sample size was chosen in each case according to experimental design based on previously published standard assays. Exact numbers are given in the figure legends and Table 1.
Data exclusions	No data was excluded from any analyses in this study.
Replication	All experiments were replicated using independent biological samples. At least 3 biological replicates were performed per conditions. All experiments were reproducible.
Randomization	Worms were randomly selected for each experiment and different conditions.
Blinding	Blinding was not used in this study.

Reporting for specific materials, systems and methods

We require information from authors about some types of materials, experimental systems and methods used in many studies. Here, indicate whether each material, system or method listed is relevant to your study. If you are not sure if a list item applies to your research, read the appropriate section before selecting a response.

Materials & experimental systems

n/a	Involved in the study	
	[x]	Antibodies
[x]		Eukaryotic cell lines
[x]		Palaeontology and archaeology
	[x]	Animals and other organisms
[x]		Clinical data
[x]		Dual use research of concern
[x]		Plants

Methods

n/a	Involved in the study	
[x]		ChIP-seq
[x]		Flow cytometry
[x]		MRI-based neuroimaging

Antibodies

Antibodies used

The following antibodies were used in this study. Polyclonal rabbit anti-GFP (TP401; 081211, Torrey Pines, 1:5000 for WB) and monoclonal anti-α-tubulin (T5168; Sigma-Aldrich; 1:2000 for WB).

Validation

Both antibodies are commercially available and validated by the manufacturer. Additionally, both are extensively used in our lab and by the scientific community. For instance in https://www.nature.com/articles/s41467-022-32377-y#additional-information.

Animals and other research organisms

Policy information about studies involving animals; ARRIVE guidelines recommended for reporting animal research, and Sex and Gender in Research

Laboratory animals

C. elegans were used through out this study. Since the worms live approximately for 3 weeks, different ages were used within this time, depending the experiment. Strains that were used, were: N2: wild-type Bristol isolate, AM141: rmIs133 [punc-54Q40::YFP], AM138: rmIs130 [unc-54p::Q24::YFP], AM140: rmIs132 [unc-54p::Q35::YFP], CL4176: smg-1(cc546) I; dvIs27[myo-3p::A-Beta (1-42)::let-851 3'UTR) + rol-6(su1006)], MAH14: daf-2(e1370) III; adIs2122 [lgg-1::GFP + rol-6(su1006)], RB1473: tli-1(ok1724) (6 times outcrossed), VP303: rde-1(ne219) V; kbIs7 [nhx-2p::rde-1 + rol-6(su1006)], NR350: rde-1(ne219)V; kzIs20 [hlh-1p::rde-1 + sur-5p::NLS::GFP], KP2018: egl-21(n476) IV, DA509: unc-31(e928) IV, CB1091: unc-13(e1091) I, ppw-1(tm5919), DCL569: mkcSi13 [sun-1p::rde-1::sun-1 3'UTR + unc-119(+)] II; rde-1(mkc36) V, NL3321: sid-1(pk3321) V, WM27: rde-1(ne219) V, NL3531: rde-2 (pk1657) I, VC1119: dyf-2&ZK520.2(gk505) III, CB193: unc-29(e193) I, CB904: unc-38(e264) I, RM1743: cha-1(md39) cho-1(tm373) IV, AY101: acIs101 [F35E12.5p::GFP + rol-6(su1006)], AU133: agIs17 [myo-2p::mCherry + irg-1p::GFP] IV, AU306: agIs44 [Pirg-4::GFP::unc-54–3'UTR; Pmyo-2::mCherry], MAH19: rrf-1(pk1417) I; myo-3(st386)V; stEx30 [myo-3p::GFP::myo-3 + rol-6 (su1006)], MAH215: sqIs11 [lgg-1p::mCherry::GFP::lgg-1 + rol-6].All strains were obtained from CGC and the S. Mitani (National Bioresource Project) in Japan, except from the ones generated in the lab (all double mutants) and the AU306 which was obtained from Read Pukkila-Worley's Lab.

Wild animals

No wild animals were used in the study.

Reporting on sex

Hermaphrodite worms were used in all experimental conditions and males were used for mating purposes.

Field-collected samples

No field-collected samples were used in this study.

Ethics oversight

No ethical oversight was required. C. elegans are invertabrates.

Note that full information on the approval of the study protocol must also be provided in the manuscript.

Plants

Seed stocks

n/a

Novel plant genotypes

n/a

Authentication

n/a
